# Polygenic risk scores as a marker for epilepsy risk across lifetime and after unspecified seizure events

**DOI:** 10.1038/s41467-024-50295-z

**Published:** 2024-07-25

**Authors:** Henrike O. Heyne, Fanny-Dhelia Pajuste, Julian Wanner, Jennifer I. Daniel Onwuchekwa, Reedik Mägi, Aarno Palotie, FinnGen, Estonian Biobank research team, Reetta Kälviainen, Mark J. Daly

**Affiliations:** 1 Hasso Plattner Institute for Digital Engineering, University of Potsdam, Potsdam, Germany; 2 Hasso Plattner Institute, Mount Sinai School of Medicine, New York, NY US; 3 Institute for Molecular Medicine Finland (FIMM), University of Helsinki, Helsinki, Finland; 4 Program for Medical and Population Genetics, Broad Institute of MIT and Harvard, Cambridge, MA USA; 5 Estonian Genome Centre, Institute of Genomics, University of Tartu, Tartu, Estonia; 6 Institute of Molecular and Cell Biology, University of Tartu, Tartu, Estonia; 7 Faculty of Life Sciences, University of Siegen, Siegen, Germany; 8 Stanley Center for Psychiatric Research, Broad Institute of MIT and Harvard, Cambridge, MA USA; 9 Kuopio Epilepsy Center, Neurocenter, Kuopio University Hospital, Member of ERN EpiCARE, Kuopio, Finland; 10 Institute of Clinical Medicine, School of Medicine, Faculty of Health Sciences, University of Eastern Finland, Kuopio, Finland; 11 Analytic and Translational Genetics Unit, Massachusetts General Hospital, Boston, MA USA

**Keywords:** Epilepsy, Genetic markers, Predictive markers, Neurological manifestations, Risk factors

## Abstract

A diagnosis of epilepsy has significant consequences for an individual but is often challenging in clinical practice. Novel biomarkers are thus greatly needed. Here, we investigated how common genetic factors (epilepsy polygenic risk scores, [PRSs]) influence epilepsy risk in detailed longitudinal electronic health records (EHRs) of > 700k Finns and Estonians. We found that a high genetic generalized epilepsy PRS (PRS_GGE_) increased risk for genetic generalized epilepsy (GGE) (hazard ratio [HR] 1.73 per PRS_GGE_ standard deviation [SD]) across lifetime and within 10 years after an unspecified seizure event. The effect of PRS_GGE_ was significantly larger on idiopathic generalized epilepsies, in females and for earlier epilepsy onset. Analogously, we found significant but more modest focal epilepsy PRS burden associated with non-acquired focal epilepsy (NAFE). Here, we outline the potential of epilepsy specific PRSs to serve as biomarkers after a first seizure event.

## Abstract (web summary)

In this study, the authors showed how common genetic factors in the form of epilepsy polygenic risk scores influence epilepsy risk across lifetime and after unspecified seizure events. This could eventually help epilepsy diagnosis.

## Introduction

Epilepsy is a serious neurological disorder characterized by unprovoked seizures, which affects up to 1% of individuals worldwide (WHO, 2019), with children and the elderly being particularly affected. Although epilepsy can be caused by acquired conditions such as stroke, tumor or head injury, most cases (ca. 70–80%) are due to genetic influences^1^, including rare and common genetic variants. Diagnosing epilepsy is often challenging^2–4^ and multiple individuals are initially misdiagnosed^5^. An epilepsy diagnosis is potentially lifesaving with a 3x elevated mortality risk in epilepsy (WHO, 2019). Epilepsy-related deaths can be prevented by antiseizure medication (ASM) which however often have adverse effects^6^. Thus, correct epilepsy diagnosis is crucial, but the most widely-used diagnostic tool in epilepsy, the electroencephalogram (EEG), has quite variable sensitivity and specificity in different clinical settings ranging about 17–58% and 70–98%, respectively^7,8^ and moderate inter-rater agreement^9^, illustrating a need for additional biomarkers^3^. Due to the great importance and challenge specific ‘first seizure clinics’ are solely dedicated to investigating an epilepsy diagnosis after a newly onset seizure^2^. Until 2014, epilepsy was defined as having two unprovoked seizures >24 h apart by the International League Against Epilepsy^10^. This definition was then extended to the following additional scenarios of having 2) a diagnosis of an epilepsy syndrome or 3) one unprovoked seizure and a probability of further seizures with a recurrence risk of at least 60% over the next 10 years^10^.

The common epilepsies can be broadly categorized into genetic generalized (GGE) and non-acquired focal epilepsies (NAFE), where the latter originate from a particular brain area^10^. First-degree relatives of patients with GGE had an 8.3-fold increased risk of developing GGE while first-degree relatives of patients with NAFE had a 2.5-fold increased risk of developing NAFE, compared to the general population, respectively^11^. In agreement, the SNP-heritability (i.e., the variance of GGE attributed to common genetic variants) is approximately 30–40%^12–14^ which is relatively high compared to other common diseases^15^. The same measure is more moderate for NAFE with SNP-heritability of about 9-16%^12,13^. Previous genome-wide association studies have shown that common variants contribute more substantially to the more common forms of epilepsy^12^. There is only a modest burden of ultra-rare genetic variants in GGE and NAFE; rare variants likely contribute only a small fraction towards their heritability^16^ and there are few Mendelian disease genes exclusively associated with them^17^.

Recent research has shown that common genetic variants with small effects on specific diseases can be combined into “polygenic” risk scores (PRSs) with high disease-specific PRSs conferring comparable risk as rare monogenic variants^18^. Thus, interest in PRSs is growing as a potential clinically important diagnostic tool^19–23^. It was recently shown that individuals with epilepsy had a significantly higher epilepsy PRS compared to unaffected controls^24,25^. However, investigating how epilepsy PRSs may predict epilepsy risk in specific clinical scenarios has so far been lacking. Here, we thus investigate how epilepsy PRSs can stratify epilepsy risk across lifetime and after unspecified seizure events.

## Results

### Electronic health records accurately represent epilepsy diagnoses

We investigated epilepsy PRSs in detailed longitudinal electronic health records (EHR) from the FinnGen project^26,27^ using FinnGen data freeze R12 (*n* = 520,105, 282,064 females) and the Estonian biobank^28^ as a validation cohort (for further study sample characteristics, see Table 1). We further explored the Bio*Me* cohort^29^ with regards to Non-European ancestries. Our phenotype data was derived from ICD codes and ASM purchases and reimbursements of official state registries spanning up to 50 years. We defined non-acquired focal epilepsy (=NAFE) by having ≥2 NAFE-specific ICD codes and genetic generalized epilepsy (=GGE) by having ≥2 GGE-specific ICD codes, respectively. Additionally, we require ≥2 ASM purchases for a NAFE or GGE category (Supplementary Fig. 1). Further details on epilepsy case definitions can be found in Supplementary Tables 1 and 2 and Methods. Sample numbers are given in Table 1. We also investigated 226 individuals with ≥2 traditional idiopathic generalized epilepsy diagnoses (=IGE)^30^ i.e. Childhood/Juvenile Absence Epilepsy (ICD 40.33/35), Juvenile Myoclonic Epilepsy (ICD 40.36) and Generalized Tonic–Clonic Seizures Alone (ICD 40.35). Individuals’ age at first epilepsy diagnosis was in line with the known age of onset of respective IGE syndromes supporting our EHR-derived diagnoses (Supplementary Fig. 2).

**Table 1:** Descriptive Statistics of main study and replication cohort

Study sample characteristics	FinnGen	EstBB (repl.)
Total participants	520,105	210,382
Female (%)	54.2	65.4
Control (*n*)	470,668	164,346
GGE (*n*)	924	275
NAFE (*n*)	5509	1233
one unspecified seizure (*n*)	2666	NA
Age Control (mean ± SD)	61.6 ± 19.7	49.1 ± 16.3
Age GGE (mean ± SD)	44.9 ± 20.6	42.3 ± 16.0
Age NAFE (mean ± SD)	61.3 ± 20.6	53.2 ± 17.4
Age at first GGE diagnosis (mean ± SD)	23.8 ± 17.4	29.5 ± 16.3
Age at first NAFE diagnosis (mean ± SD)	45.9 ± 22.9	41.7 ± 19.6
Follow-up time after unsp. seizure (mean ± SD)	14.8 ± 11.2	NA
Ancestry (continental)	European	European

*FinnGen* FinnGen study (Finland), *EstBB* Estonian biobank (Estonia), *repl* replication cohort. All ages and times are given in years.

### Epilepsy PRS is most elevated in GGE, specifically IGE, with the same effect sizes as in clinically curated epilepsy cohorts

**Fig. 1:**
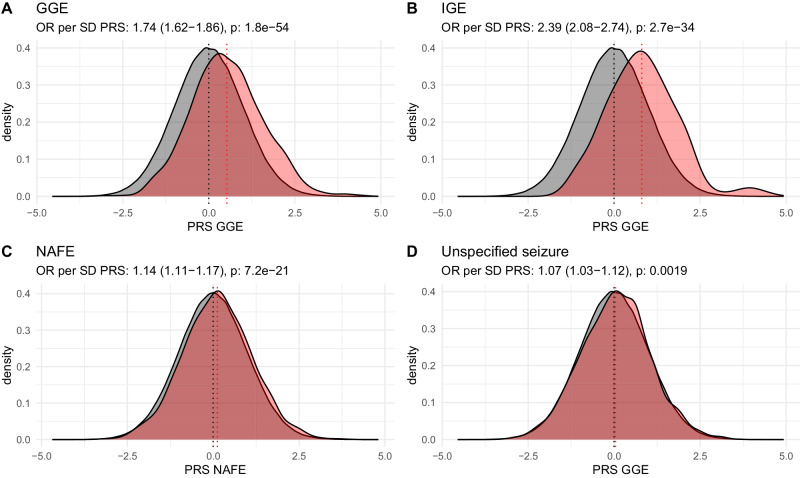
Epilepsy PRS of epilepsy cases (red) compared to population controls (gray) (*n* = 273,974) (density curves). **A** PRS_GGE_ of GGE (*n* = 924) compared to controls. **B** PRS_GGE_ of IGE (*n* = 226) compared to controls. **C** PRS_NAFE_ of NAFE (*n* = 5509) compared to controls. **D** PRS_GGE_ of unspecified seizure without epilepsy (*n* = 2485) compared to controls. On top of each panel: odds ratios (95%- confidence intervals in brackets) and *p*-values of standard deviation increase of epilepsy PRS on case versus control status. Mean of the PRS distributions is shown as vertical dotted lines. Method: logistic regression. OR; odds ratio, *p*; *p*-value.

**Fig. 2:**
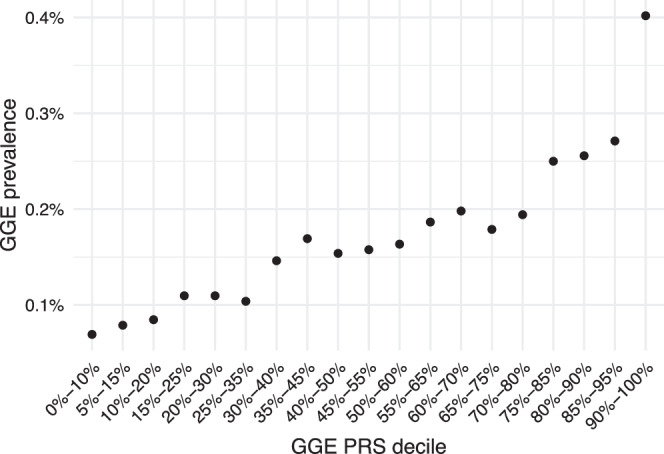
Correlation of PRS_GGE_ decile and GGE prevalence. The bins of adjacent PRS deciles are overlapping, as labeled. Data points are also provided as a Source Data file.

We then calculated epilepsy PRSs to determine individuals’ genetic burden for epilepsy. Here, we used the International League Against Epilepsy (ILAE) genomewide association study’s (GWAS) 2023 summary statistics^13^ as discovery data, i.e. to determine which genetic variants increase or decrease epilepsy risk. We then summed 1000 s of genetic risk/protective variants for epilepsy with individually small effects into a single epilepsy PRS per individual. Here, we constructed separate focal epilepsy PRS (PRS_NAFE_) and generalized epilepsy PRS (PRS_GGE_). We found a significant elevation of PRS_GGE_ in 924 individuals with GGE (Fig. 1A) which was particularly pronounced in IGE (see also next paragraph and Fig. 1B). We also found a significant elevation of PRS_NAFE_ in 5509 individuals with NAFE (Fig. 1C), but no significant elevation of PRS_GGE_ in individuals with unspecified seizures (Fig. 1D). Similarly, we found a high correlation (Pearson’s correlation coefficient = 0.91, *p*-value = 4 × 10^−8^) between PRS_GGE_ decile and GGE prevalence in our data (Fig. 2). Overall, we can thus confirm previous studies that used PRS as a marker for genetic liability of common epilepsy types. Importantly, we find very similar respective effect sizes of PRS_GGE_ and PRS_NAFE_ on GGE and NAFE as reported in previous cohorts from Epi25 or the Cleveland Clinic^24^ when using the same GWAS^12^ in an earlier version of the manuscript^31^ or the updated GWAS^13^ (see Table 2). We thus consider it likely that the epilepsy phenotypes in our biobank data are comparable to the phenotypes curated according to clinical criteria in these cohorts.

**Table 2:** Enrichment of epilepsy cases in individuals with increasing epilepsy PRS

PRS/ phenotype	PRS bin	OR	5%-CI	95%-CI	*p*-value	cases in bin	controls in bin	sensitivity	specificity
GGE	20%	2.39	2.07	2.75	3E-33	342	94376	0.36%	99.85%
GGE	5%	3.20	2.61	3.89	6E-30	129	23530	0.55%	99.82%
GGE	2.50%	3.86	2.99	4.91	1E-26	82	11756	0.69%	99.82%
GGE	1%	4.24	2.90	5.98	5E-15	39	4676	0.83%	99.81%
GGE	0.50%	4.64	2.73	7.33	9E-10	22	2333	0.93%	99.81%
NAFE	20%	0.85	0.79	0.91	2E-06	1184	94376	1.14%	98.76%
NAFE	5%	1.35	1.20	1.52	3E-07	339	23530	1.42%	98.86%
NAFE	2.50%	1.46	1.25	1.71	2E-06	181	11756	1.52%	98.85%
NAFE	1%	1.65	1.29	2.07	4E-05	80	4676	1.68%	98.85%
NAFE	0.50%	1.43	0.97	2.02	0.053	35	2333	1.48%	98.84%

Odds ratios for epilepsy case status were calculated comparing individuals in bins of the top 0.5%, 2.5%, 5% and 20% of epilepsy PRS with the remainder of the cohort as done in ref. ^24^. PRS was matched to epilepsy type, i.e. the effect of PRS_NAFE_ on NAFE case status, and the effect of PRS_GGE_ on GGE case status was estimated. There we in total *n* = 924 GGE and 5509 NAFE cases and *n* = 470,551 controls. Method: logistic regression. Covariates: sex, birth year, age at last follow up, first 10 PCs of ancestry, genotyping batch.

*OR* odds ratio, *CI* confidence interval. *p*-values are not adjusted for multiple comparisons. Dataset: FinnGen.

### High PRS is associated with epilepsy across lifetime and after unspecified seizure events

**Fig. 3:**
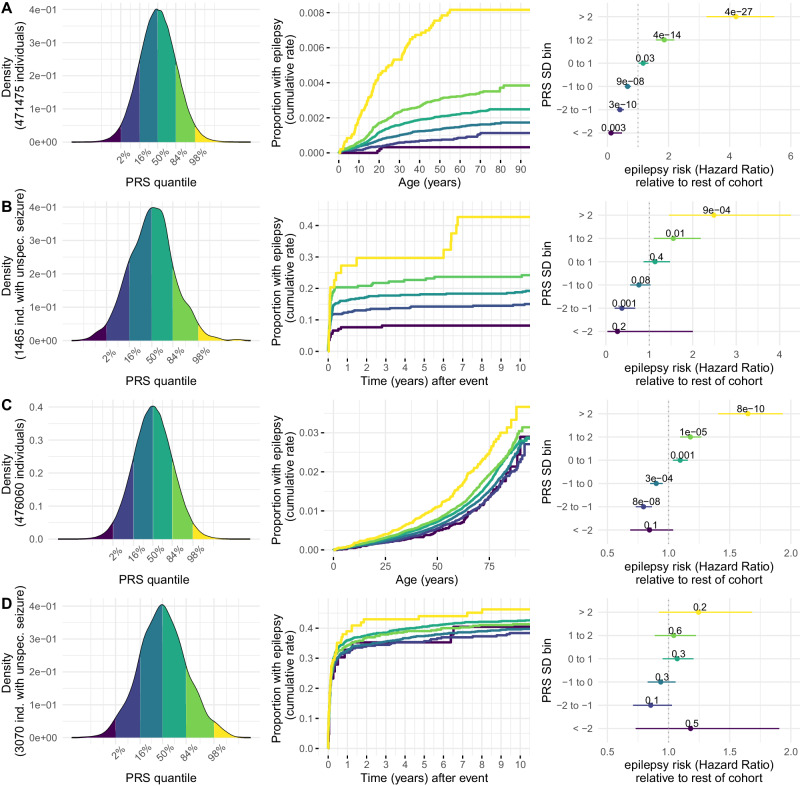
Epilepsy PRS as a marker for epilepsy risk across lifetime and after unspecified seizure events. **A** GGE (*n* = 924) across lifetime, (**B**) GGE (*n* = 266) after an unspecified seizure, (**C**) NAFE (*n* = 5509) across lifetime, (**D**) NAFE (*n* = 1290) after an unspecified seizure. In (**A**, **B**), we investigate PRS_GGE_, in (**C**, **D**), PRS_NAFE_. In each panel, on the left are density curves that display how samples are partitioned into six bins of PRS standard deviations. Survival curves in the middle give the cumulative epilepsy incidence (*y*-axis) across time (x-axis [years]) stratified for epilepsy PRS bins. In (**B**), the two lowest PRS bins are fused in the survival curve as epilepsy case counts were too low. The rightmost forest plots show epilepsy risk of each epilepsy PRS bin compared with the rest of the cohort. Here, the point estimates represent hazard ratios (method: cox proportional hazard’s model), error bars show the 95%-confidence intervals (CIs). Summary statistics are provided as a Source Data file.

We next investigated the effect of epilepsy PRS on epilepsy rates across lifetime, separately for PRS_GGE_ and PRS_NAFE_. We stratified our cohort into bins of epilepsy PRS standard deviations (SD) (Fig. 3) and compared the cumulative epilepsy incidence in each SD bin to the rest of the cohort (for increased power). Individuals with a PRS_GGE_ > 2 SD (ca. 2% of the cohort) had a more than 4-fold lifetime risk of developing GGE than the rest of the cohort (Hazard ratio [HR]: 4.2, Confidence Interval [CI]: 3.2-5.4, *p*-value: 4 × 10^−27^, method: cox proportional hazard model^32^ [coxph], Fig. 3, panel A). The epilepsy risk decreased proportionally with the decreasing PRS_GGE_ SD bin. Overall, the HR increased by 1.73 per increased SD of PRS_GGE_ (Table 3, 95%-CI 1.62–1.86, *p*-value = 8 × 10^−55^). When restricting to IGE the HR per PRS_GGE_ SD was 2.4 (95%-CI 2.1–2.7, *p*-value 2 × 10^−34^). Individuals with a PRS_GGE_ > 1 SD had a HR of 12.1 for IGE compared to those with PRS_GGE_ < −1 SD (95%-CI 6-25, *p*-value 3 × 10^−11^, IGE rate in PRS_GGE_ < −1 SD: 8/75,114, IGE rate in PRS_GGE_ > 1 SD: 88/75,505). PRS discriminated GGE cases versus controls with a concordance index (C-index) of 0.64 (95%-CI 0.61–0.68) adjusting for the same covariates (birth year, sex, batch and PCs). Overall, we thus showed PRS_GGE_ as a significant biomarker for lifetime epilepsy risk.

**Table 3:** Effects of PRS on epilepsy risk in biobanks FinnGen and Estonian biobank

				HR per SD PRS (95%- CI, *p*-value)	
Epilepsy	PRS	*n* epilepsy (FinnGen)	Incidence in time period	FinnGen	EstBB
GGE	GGE	924	Lifetime	1.73 (1.62–1.86), 8 × 10^−55^	1.58 (1.40–1.78), 2 × 10^−13^
NAFE	NAFE	5509	Lifetime	1.14 (1.10–1.17), 4 × 10^−21^	1.11 (1.05–1.18), 3 × 10^−4^
IGE	GGE	226	Lifetime	2.38 (2.07–2.74), 2 × 10^−34^	–
GGE	GGE	266	unspecified seizure (10 yrs)	1.55 (1.35–1.78), 5 × 10^−10^	–
NAFE	NAFE	1290	unspecified seizure (10 yrs)	1.07, (1.02–1.2), 0.01	–

Epilepsy risks are given as hazard ratios (HR) for epilepsy per SD increase of epilepsy PRS. Method: Cox proportional hazards model. *CI* confidence interval, *FinnGen* FinnGen study (Finland), *EstBB* Estonian biobank (Estonia), unspecified seizure (10 yrs); 10 years after an unspecified seizure (age <40 years old for GGE and <60 years old for NAFE). Sample sizes in EstBB: *n* = 275 (GGE), *n* = 1233 (NAFE).

However, the absolute risk of developing epilepsy is small across lifetime (<1%, see Fig. 3A/C), even for individuals with high epilepsy PRS. Lifetime risk prediction is thus less clinically meaningful. When considering the subset of individuals that were diagnosed with an unspecified seizure corresponding to ICD code R56.8/7803A at an age <40 years their absolute risk for GGE increased compared to baseline (Fig. 3B). Within 10 years after the unspecified seizure, the GGE rate reached 42% in > 2 SD PRS_GGE_ compared to 4% in <−2 SD PRS_GGE_ (or 27% in > 1 SD PRS_GGE_ versus 8% in < −1 SD PRS_GGE_). PRS_GGE_ affected relative epilepsy risk similarly after an unspecified seizure (HR per PRS_GGE_ SD: 1.5, 95%-CI: 1.3–1.8, *p*-value = 1 × 10^−9^, C-index 0.60, 95%-CI 0.53-0.67) as across lifetime. Similarly, PRS_NAFE_ had a significant but more modest effect on NAFE cumulative lifetime incidence (HR per PRS_NAFE_ SD: 1.13, 95%-CI: 1.09-1.17, *p*-value = 3 × 10^−10^) and after unspecified seizure (HR per PRS_NAFE_ SD: 1.075, 95%-CI: 1.014–1.14, *p*-value = 0.02), in line with a lower heritability of focal epilepsy (Fig. 3C, D). In addition, we tested the effect of a PRS_all-epilepsy_ computed from a GWAS of all epilepsy phenotypes, including unclassified epilepsy, on lifetime epilepsy. Unfortunately, we found only limited association with lifetime risk of GGE, NAFE or any epilepsy (Supplementary Table 3).

We replicated analyses of PRS_GGE_ effects across lifetime in the Estonian biobank^28^ (Estonia, European ancestry, Supplementary Fig. 3), obtaining similar estimates (see Table 3) and thus validating our results. We further explored the effects of PRS_GGE_ in individuals with diverse ancestries in the Bio*Me* biobank (Supplementary Fig. 4, Supplementary Note), a biobank that links genetic and EHR data for more than 30,000 individuals from diverse ancestral and cultural backgrounds recruited primarily in the Mount Sinai Health System in New York City. While the effect of PRS_GGE_ in Bio*Me* followed similar trends, our analyses were underpowered and thus did not reach significance. Further analyses are needed to investigate the portability of epilepsy PRS effects to other ancestry groups.

### Epilepsy PRS has sex-specific effects on epilepsy subtypes

In other diseases than epilepsy, studies previously reported sex-specific PRS effects and larger effects of PRS on disease in earlier age groups^33^. Thus, we sought to investigate the effect of age at onset and sex on PRS effects on epilepsy. We found a significant interaction of sex and PRS_GGE_ on GGE case (*n* = 924) status (cox model *p*-value 0.002, regression *p*-value 0.02). So we next investigated the effect of PRS on lifetime epilepsy separately for men and women. PRS_GGE_ had a larger influence on lifetime GGE in females (HR_female_ per PRS SD: 1.9, 95%-CI 1.7–2.0, *p*-value = 1 × 10^−47^, *n*_GGE_ = 543) than in males (HR_male_ per PRS SD: 1.5, 95%-CI 1.3–1.7, *p*-value = 2 × 10^−11^, *n*_GGE_ = 381, Supplementary Fig. 5). We further found a higher prevalence of epilepsy in females, specifically with onset in the teenage—young adult range (Supplementary Fig. 6). Exploring sex-specific effects on specific epilepsy types we found no significant effect of sex (*p* = 0.4) nor PRS*sex interaction (*p* = 0.7) in IGE (*n* = 226) but found a significant effect of sex (*p*-value 7 × 10^−4^) and PRS*sex interaction (1 × 10^−3^) in non-IGE GGE (*n* = 657). Similarly, the effect of PRS_GGE_ on non-IGE GGE was substantially higher in females (HR_female_ 1.78, 95%-CI 1.60–1.99, *p*-value 5×10^−26^, HR_male_ 1.32, 95%-CI 1.16–1.50, *p*-value 2 × 10^−5^) while it was quite comparable for IGE (HR_female_ 2.35, 95%-CI 1.16–1.60, *p*-value 1 × 10^−25^; HR_male_ 2.57, 95% CI 1.92–3.44, *p*-value 2 × 10^−10^). We also found a significant interaction of sex and PRS_NAFE_ on NAFE (*p*-value 0.008) with slightly higher PRS_NAFE_ effects on NAFE in females (HR_male_: 1.10, 95%-CI 1.06–1.15, *p*-value = 2 × 10^−6^; *n* = 2706, HR_female_: 1.18, 95%-CI 1.13–1.22, *p*-value = 2 × 10^−17^, *n* = 2806).

### Epilepsy PRS has a larger effect when epilepsy onset is earlier

We further explored whether epilepsy PRS effects were potentially different for different ages of epilepsy onset. We thus divided our cohort into quintiles of age at first epilepsy diagnosis and found significant effects of PRS_GGE_ on GGE and of PRS_NAFE_ on NAFE case status in all age at onset bins except GGE onset > 60 years and NAFE onset > 80 years (method logistic regression, see Supplementary Table 4). We found the largest effects of PRS_GGE_ when individuals had earlier ages at first diagnosis, e.g. for GGE effects were largest at onset 0-20 (OR 1.9, 95%-CI 1.7–2.1, *p*-value 9 × 10^−38^) and of PRS_NAFE_ on NAFE at onset 20-40 years (OR 1.21, 95%-CI 1.14–1.28, *p*-value 4 × 10^−10^). This is in line with other illnesses^33^. We next investigated, if the large genetic influences on IGE described in the paragraph above could be explained by a higher proportion of individuals with younger age at epilepsy onset in the IGE group. So within the GGE group, we compared the effect of PRS_GGE_ on IGE versus non-IGE and still found a higher effect of PRS_GGE_ on IGE even when accounting for age at first epilepsy diagnosis (OR 1.58, 95%-CI 1.29–1.95, *p*-value 2 × 10^−5^).

### PRS_GGE_ is specifically associated with GGE while PRS_NAFE_ is more heterogeneous

**Fig. 4:**
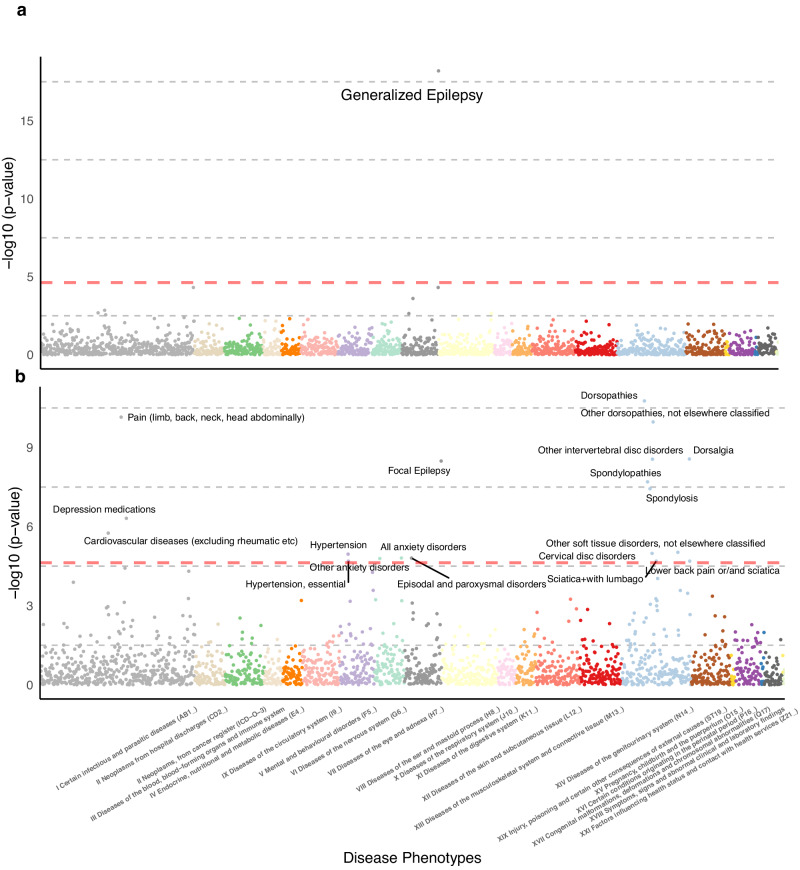
Phenome-wide association study testing the effect of epilepsy PRS on 2139 distinct disease phenotypes in FinnGen. In (**a**) we display a PheWAS of PRS_GGE_. GGE, here labeled as ‘Generalized Epilepsy’, is the only phenotype that is significant after Bonferroni correction. In (**b**) we display a PheWAS of PRS_NAFE_. In both panels, the phenotypes are grouped and colored by clinical field (*x*-axis). The y-axis shows the -log10 *p*-value of the association (method: logistic regression). The dashed orange horizontal line at *p*-value 2.3 × 10^−5^ is the significance threshold after multiple testing correction (Bonferroni). Summary statistics are provided as a Source Data file.

We aimed to investigate the phenotypes associated with a genetic epilepsy liability that are *not* epilepsy, in a hypothesis-free approach to elucidate whether genetic factors influence GGE/NAFE in a disease-specific manner. We thus performed a phenome-wide association study (PheWAS) testing the effect of PRS_GGE_ and PRS_NAFE_ on 2139 distinct disease phenotypes (method: logistic regression, FinnGen data freeze: R6, GWAS: ILAE 2018^12^, Fig. 4). GGE (labeled as ‘Generalized Epilepsy’) is the only phenotype that is significantly affected by PRS_GGE_ after Bonferroni correction. We thus argue that PRS_GGE_ is very specifically associated with GGE increasing its potential diagnostic utility. While PRS_NAFE_ is expectedly associated with NAFE, multiple other phenotype associations are unexpected. The most significant ones are related to back pain, but also include hypertension, cardiovascular disease and depression medications, with lower significance. We tested the genetic correlation of NAFE and the 19 traits that were significant in our PheWAS (method: LD score regression^34,35^, Supplementary Fig. 7). After multiple testing correction none remained significant. However, phenotypes ‘other anxiety disorders’ (*r*_*g*_ = 0.54, *p*-value = 0.02), ‘all anxiety disorders’ (*r*_*g*_ = 0.44, *p*-value = 0.02) and ‘depression medications’ (*r*_*g*_ = 0.33, *p*-value = 0.04) were genetically correlated with nominal significance.

## Discussion

The diagnosis of epilepsy is an important yet challenging clinical task; thus the need for novel biomarkers remains high. Recent studies demonstrated a genetic burden in the form of an elevated PRS_GGE_ in epilepsy cases versus controls^24,25^ which we replicate in our data. The effect of PRS_GGE_ has however not been studied outside the case control setting. Here, we investigate the effect of PRS_GGE_ longitudinally; on lifetime epilepsy, on epilepsy after an unspecified seizure event and on 1000 s of disease endpoints in other clinical areas.

In this study, we could demonstrate that common genetic variants, in the form of PRS_GGE_ have a significant quantitative effect on GGE lifetime cumulative incidence that we could reproduce in another biobank with hazard ratios of 3-4 for the upper tails of the PRS_GGE_ distribution in line with previous studies^24^ and after unspecified seizure events. Predictions are modest with C-indices of ca. 0.6, but comparable to the performance of models using clinical variables (C-indices in similar ranges of ca. 0.6 reported in the MESS trial^4^ or in EEG studies^36,37^). Thus, we expect PRSs to have potential utility as a supportive but not standalone tool. In our data, the effect of PRS_NAFE_ on NAFE across lifetime was also significant but more modest than for PRS_GGE_ and GGE, in line with other studies.

We were surprised to find that the effect of PRS_GGE_ on GGE was substantially larger in females than males which was not previously reported. Previous studies reported a higher incidence of GGE in women^38,39^ which we also observed in our cohort. These could be caused by a different epilepsy susceptibility in males and females mediated by biological or environmental sex-specific factors. This is likely not caused by different pathomechanisms as a recent study found a high correlation of genetic effects on epilepsy in males and females^13^. However, we find sex-specific PRS effects predominantly for non-IGE suggesting sex-specific genetic factors may differentially influence risk for specific epilepsy subtypes. Thus, further research is needed to elucidate how genetic factors may differently influence epilepsy between sexes.

The effect of PRS_GGE_ on GGE was quite specific with no significant effects on other diseases. However, we did not test the effects on non-disease phenotypes. Previously, high PRS_GGE_ and high PRS_NAFE_ were both associated with low educational attainment and neuroticism-related personality traits^40^ which could result from epilepsy or side effects of ASMs or may also be pleiotropic effects. Apart from NAFE, PRS_NAFE_ had effects on other diseases including back pain, which was not previously reported; and anxiety/depression-related traits. Here, nominal significant genetic correlations of NAFE with anxiety disorders and depression medications are in line with previous reports in the UK biobank that individuals with high PRS_NAFE_ but without a NAFE diagnosis had more likely experienced anxiety or depression^40^ pointing to a potential pleiotropic effect. Co-morbidities of chronic pain and depression have been previously reported^41^.

We see the highest potential clinical utility of epilepsy PRS in patient groups with a high absolute risk of having epilepsy such as after an unspecified seizure event. Current clinical guidelines require at least one unprovoked seizure and at least a 60% chance of a second seizure to diagnose epilepsy^10^. In a clinical setting, the diagnosis is often not as quantifiable as the definition suggests and is heavily dependent on clinical expertise. We find, as an example, that individuals with a PRS_GGE_ > 2 SD have a > 3x increased risk of being diagnosed with GGE than the rest of the population. This includes individuals with unspecified seizure events who are at elevated risk for a later epilepsy diagnosis. After the exclusion of reversible causes for their unspecified seizure, a high PRS_GGE_ could support stratifying groups at risk for a second seizure in conjunction with an EEG while other biomarkers are currently sparse^3^. Other recent studies suggest that PRS_GGE_ have additional value to the information of family history^42,43^. Practically, genetic testing is regularly done in pediatric epilepsy and generation of PRS could thus potentially be integrated in an existing workflow. Here, integrating PRS with rare variants could also improve disease prognosis as genetic background has been shown to influence how severely carriers of genetic variants with large disease effects^44^ such as Dravet syndrome^45^ are affected. Another advantage is a high cost-effectiveness as PRS can be generated from genotype data that can also be repurposed from other disease areas^23^.

Our study has several limitations. We have conducted most of our analyses in cohorts with European ancestry. As has been previously described for other diseases, the predictive ability of polygenic risk scores is heavily dependent on genetic ancestry^14^. While the effect of PRS_GGE_ on epilepsy showed similar trends in the primarily non-European Bio*Me* cohort sample sizes remained prohibitive. Further studies in diverse populations are thus needed. Another limitation is that our phenotype data is derived from EHRs. We can thus not verify how many epilepsy cases have been confirmed by epileptologists. However, we obtain similar PRS effect sizes as in clinical cohorts^24^, which thus validates our case definitions by combining EHR diagnoses with ASM purchase and reimbursement data. The central registry of Finnish EHR data have the unique advantage that reimbursements for ASMs are always based on a certificate made by a neurologist. In addition, while we excluded individuals that were also part of the discovery GWAS we did not have the option to directly compare individual-level data between the discovery GWAS and our validation cohorts. We could thus not control for any potential relatedness between the cohorts with the potential to inflate our results^46^.

Our data thus proposes an interesting potential for epilepsy PRS, specifically for PRS_GGE_, as a biomarker for epilepsy risk where it could—combined with clinical markers such as the EEG—improve epilepsy risk prediction. Our data outlines how this could be specifically useful in situations of elevated epilepsy risk such as an unspecified seizure event. Ultimately, this needs to be investigated in a clinical setting.

## Methods

This study complies with all relevant ethical regulations; the Ethics Committee of the Hospital District of Helsinki and Uusimaa approved the study protocol for FinnGen (Nr HUS/990/2017), the Estonian Committee on Bioethics and Human Research for Estonian biobank (protocol 1.1-12/624) and the Icahn School of Medicine at Mount Sinai Institutional Review Board (IRB; approval STUDY-19-00951) for Bio*Me*.

### Data and definition of epilepsy cases and controls

Here, we define epilepsy case and control status from detailed longitudinal EHR of the FinnGen project^27^ using data freeze R12 as a main cohort (*n* = 520,105) and Estonian biobank^28^ as an additional validation cohort (*n* = 210,382). We use phenotype data derived from official state registries. These include 9,313 individuals with epilepsy ICD codes, 2,485,702 ASM purchases and 12,695 ASM reimbursements of ATC codes N03A*. We list an overview of case definitions and numbers in Supplementary Table 1. 94.7% of individuals with ≥ 2 generalized seizure ICD codes and 93.7% of individuals with ≥ 2 focal seizure ICD codes purchased ≥ 2 ASMs, while only 16.4% of individuals without epilepsy diagnoses purchased ≥ 2 ASMs (see Supplementary Fig. 1). This cross-validates our EHR data.

Reimbursement rights for epilepsy are derived from the Social Insurance Institution of Finland (KELA), Finland’s national authority. All persons with newly diagnosed epilepsy are eligible for ASM reimbursement, which is also routinely applied for, necessitating a detailed statement by a neurologist and investigations at a specialist clinic. The statement is checked and approved by specialist physicians at the reimbursement institution KELA before the right is granted. Epilepsy diagnoses in Finland are made according to national guidelines, which are updated according to ILAE epilepsy definitions.

We thus chose the following criteria to define GGE:

- at least two ICD codes of G40.3 (“Generalized idiopathic epilepsy […]”) or corresponding ICD9 codes (Supplementary Table 2) and at least two purchases of ASMs (as defined by N03* ATC codes).

We chose the following criteria to define NAFE:

- at least two ICD codes of G40.0, G40.1, G40.2 (“Localization-related (focal)(partial) […] epilepsy […]”) or corresponding ICD9 diagnoses (Supplementary Table 2) and at least two purchases of ASMs.
- excluded possible structural etiology of focal seizures such as stroke, brain tumor, CNS infection and CNS injury (for ICD codes see Supplementary Table 2). Here, we only excluded individuals if they had their first seizure event within one year after the brain-related potential epileptogenic event.

For 1008 individuals with both focal and generalized epilepsy codes we applied the following additional criteria for a GGE diagnosis:

- more generalized than focal epilepsy codes AND
- most frequent ICD code is a generalized epilepsy code AND
- no reimbursement category of focal epilepsy.

We used the same criteria vice versa to define NAFE among individuals with focal and generalized epilepsy codes.

We defined idiopathic generalized epilepsy (IGE) according to ILAE^30,47^ by at least two ICD codes of 40.33 (Childhood Absence Epilepsy), 40.34 (Generalized Tonic–Clonic Seizures Alone, here using the ICD Code of the formerly known term Generalized Tonic–Clonic Seizures on Awakening) 40.35 (Juvenile Absence Epilepsy), 40.36 (Juvenile Myoclonic Epilepsy). See Supplementary Fig. 2 for age at first diagnosis.

We used individuals without epilepsy-related diagnoses as controls. We excluded individuals who purchased ASMs from the control group.

For the analysis of GGE incidence following an unspecified seizure event, we used the same diagnosis of GGE as described above. We defined an unspecified seizure event with an ICD code of R56.8/7803 A (‘unspecified convulsions’). From the group with a single unspecified seizure event we excluded individuals

- with any other epilepsy-related diagnoses (G40/G41 ICD codes) AND
- who purchased or reimbursed ASMs within two years before up to 10 years after event AND
- who were at any time diagnosed with alcohol-related ICD codes OR who had multiple unspecified seizure events (to exclude potential alcohol withdrawal seizures).

When individuals had 2 seizure diagnoses on the same day we counted them as one seizure event as they most likely represent two labels of the same event. When the 2 seizure diagnoses had discordant ICD labels we labeled them according to the most specific ICD code. (As an example, individuals with diagnoses of unspecified seizure and generalized epilepsy on initial presentation would be classified as diagnosed with ‘generalized epilepsy’ on initial presentation.)

We defined epilepsy cases similarly in the validation cohort Estonian biobank, with the only exception that instead of using the reimbursement data to differentiate between NAFE and GGE in individuals who had both focal and generalized epilepsy codes, we used prescription data. Specifically, we excluded individuals as GGE cases if they had any ASM prescriptions that listed NAFE as a reason for the prescription and vice versa. We performed the unspecified seizure event analysis only in FinnGen where we had a sufficient sample size.

Importantly, we find very similar respective effect sizes of PRS_GGE_ and PRS_NAFE_ on GGE and NAFE as reported in previous cohorts from Epi25 or the Cleveland Clinic^24^ when using the same GWAS^12^ in a previous version of the manuscript^31^ or the updated GWAS^13^ (see Table 2). We acknowledge, that PRS effects in our cohort may not be directly comparable as we are using a different PRS calculation method (using all 835 K weighted SNPs^48^ instead of classic clumping and using SNPs <*p*-value threshold 0.5^24^). However, differences in phenotype definitions have been reported to have larger effects than differences in PRS methods^49^, specifically for epilepsy^13^. We thus consider it likely that the epilepsy phenotypes in our biobank data are comparable to the phenotypes curated according to clinical criteria in these cohorts.

### Calculation of polygenic risk scores

We calculated epilepsy PRS with the method PRS-CS^48^. Here, we used the summary statistics from the ILAE GWAS 2023^13^ and ILAE GWAS 2018^12^ (only PheWAS analyses and analyses in the BioMe cohort) as discovery data, i.e. to determine which genetic variants increase or decrease epilepsy risk. We constructed separate focal PRS (PRS_NAFE_) and generalized epilepsy PRS (PRS_GGE_). The ILAE 2023 GWAS contained the FinRisk cohort (n > 40k controls, part of FinnGen). We therefore excluded 25,405 FinRisk samples from the controls of our study to avoid overlap with the GWAS discovery cohort. The Finnish GenEpa cohort part of both GWAS was not part of FinnGen. We applied the PRS-CS-auto algorithm to infer posterior effect sizes for the variants for PRS calculation. PRS-CS-auto learns the model’s global scaling parameter ϕ from the data. We used data from the 1000 Genomes^50^ as a reference panel for linkage disequilibrium. We then weighted and summed all available genetic variants that confer either risk for or protection from epilepsy into a single epilepsy PRS per individual using the PLINK–score command^51^. The PRS-CS pipeline in FinnGen is described in more detail at https://github.com/FINNGEN/CS-PRS-pipeline. We provide the PRS weights file as Supplementary Data 1.

In FinnGen and EstBB, we restricted our analysis to individuals with European ancestry, while we additionally included African and American continental ancestry groups in the Bio*Me* cohort (Supplementary Note). We inferred population labels based on principle component analysis of the genotype data as described previously^27,28^.

### Statistical analyses

We used the R programming language for all statistical analyses. Pipelines for parallel computing were created using Cromwell-29 and 31 and Wdltool-0.14. Statistical analyses and figures were done using different version of R packages ggplot2^52^, data.table, plyr, survminer, survival, tidyr and Rutils.

In all analyses including PRSs namely logistic regression, survival analyses and concordance index calculations, we included the following covariates: the first 10 principal components of genetic markers (10 PCs) as a proxy for population substructure and ancestry, genotyping batch (only in the FinnGen cohort), sex, birth year, age at last follow up. For analyses that included only individuals with seizures, we included age at the first epilepsy diagnosis as a covariate instead of age at the last follow-up. We tested PRS_GGE_, PRS_NAFE_ and PRS_all-epilepsy_ as indicated in the results of the manuscript.

All statistical tests were conducted as two-sided hypotheses without assuming a specific direction. No statistical method was used to predetermine sample size, instead the maximum number of available samples from the respective studies were used.

In the PheWas, we defined independent diseases when for any disease category not more than 40% of affected individuals are listed in any other disease category.

We performed survival analyses using the Cox Proportional-Hazards model (Cox-PH)^32^. Follow-up starts at birth and ends at the age of first epilepsy diagnosis (for individuals with epilepsy), age at last record available in the EHR or death, depending on what happened first. We also performed survival analyses in individuals with an unspecified seizure. Here, follow-up started at the age of the unspecified seizure and ended at the age of first epilepsy diagnosis, age at last record available in the EHR, death or after 10 years, depending on what happened first. We tested for sex differences by including an interaction term of PRS x sex in the Cox-PH model. We used the first 10 PCs, genotyping batch, sex, birth year and age at last follow up as covariates in all survival analyses. We did not exclude individuals that were related as we found in sensitivity analyses of a different project that this did not influence the PRS effect on disease^53^. As an additional check, we repeated our survival analysis after excluding 320,226 related individuals (corresponding to kinship values > 0.04 and > 3rd degree relatedness using the software KING^54^) from the FinnGen data. The effect sizes of PRS on lifetime GGE risk (method: cox model, for IGE: HR of 2.39 per SD PRS, 95%-CI 2.1–2.7, *p*-value 2 × 10e−34, for GGE: HR of 1.74 per SD PRS, 95%-CI 1.6–1.9, *p*-value 1 × 10e−53) remained almost identical. This may be expected since we found few related individuals among GGE (18 out of 924) and IGE (6 out of 226) cases. We excluded sex in sex-specific survival analyses. We included age at first unspecified seizure in survival analyses of individuals with an unspecified seizure.

### Reporting summary

Further information on research design is available in the Nature Portfolio Reporting Summary linked to this article.

### Supplementary information

Supplementary Information

Peer Review File

Description of Additional Supplementary Files

Supplementary Data 1

Reporting Summary

### Source data

Source Data

## Data Availability

All results described in this manuscript can be found in the (Supplementary) Tables. A full list of FinnGen endpoints for release 12 is available at www.finngen.fi/en/researchers/clinical-endpoints. Individual level data in this study are not publicly available due to legal and privacy limitations, but they can be accessed through individual participating biobanks. The FinnGen data may be accessed through Finnish Biobanks’ FinBB portal (www.finbb.fi; email: info.fingenious@finbb.fi). Researchers interested in Estonian Biobank can request access at https://www.geenivaramu.ee/en/access-biobank. For access to data from Bio*Me* biobank, please read here (https://icahn.mssm.edu/research/ipm/programs/biome-biobank). For questions, please reach out to biomebiobank@mssm.edu. Source data in the form of summary statistics are provided with this paper. Source data are provided with this paper.

## Code availability

Code for FinnGen core analyses is available at https://github.com/FINNGEN/.
