# Supplementary material for: Polygenic risk scores as a marker for epilepsy risk across lifetime and after unspecified seizure events
Source: Nat Commun. 2024 Jul 25;15:6277. doi: 10.1038/s41467-024-50295-z (PMC11272783; doi:10.1038/s41467-024-50295-z)
Supplement: Supplementary file 1 — Supplementary Information [file 41467_2024_50295_MOESM1_ESM.pdf]

Supplementary Figures

Supplementary Figure 1. Antiseizure medication (ASM) purchases. The three barplots show ASM purchases of individuals with at least two epilepsy diagnosis codes of GGE (n=1,336) or NAFE (n=7,929), respectively, and without epilepsy diagnosis codes (‘control’, n=520,105). Counts are grouped for individuals who made 0 (blue), 1 (red) and ≥2 (yellow) ASM purchases, respectively.

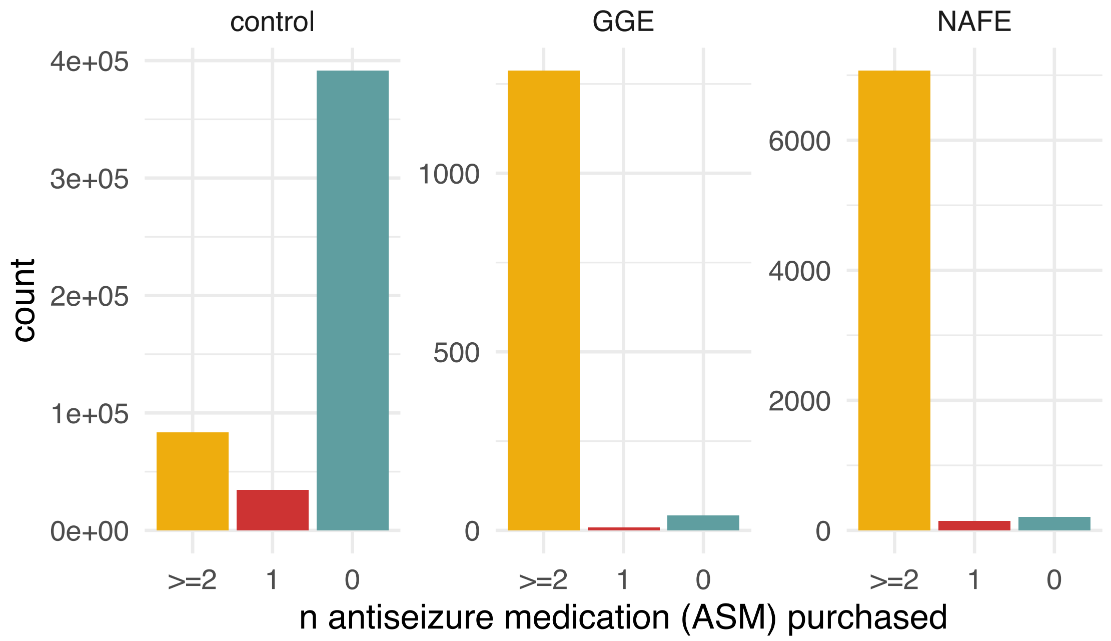

Supplementary Figure 2. Age at IGE diagnosis. Age at first diagnosis of individuals with at least 2 diagnoses of idiopathic generalized epilepsy diagnoses (=IGE) [1]. The four panels show age at diagnosis of individuals with most frequent ICD codes of Childhood Absence Epilepsy (G40.33, n=32), Generalized Tonic–Clonic Seizures on Awakening (GTCA, *now:* Generalized Tonic–Clonic Seizures Alone) (G40.34, n=10), Juvenile Absence Epilepsy (G40.35, n=43) Juvenile Myoclonic Epilepsy (G40.36, n=141).

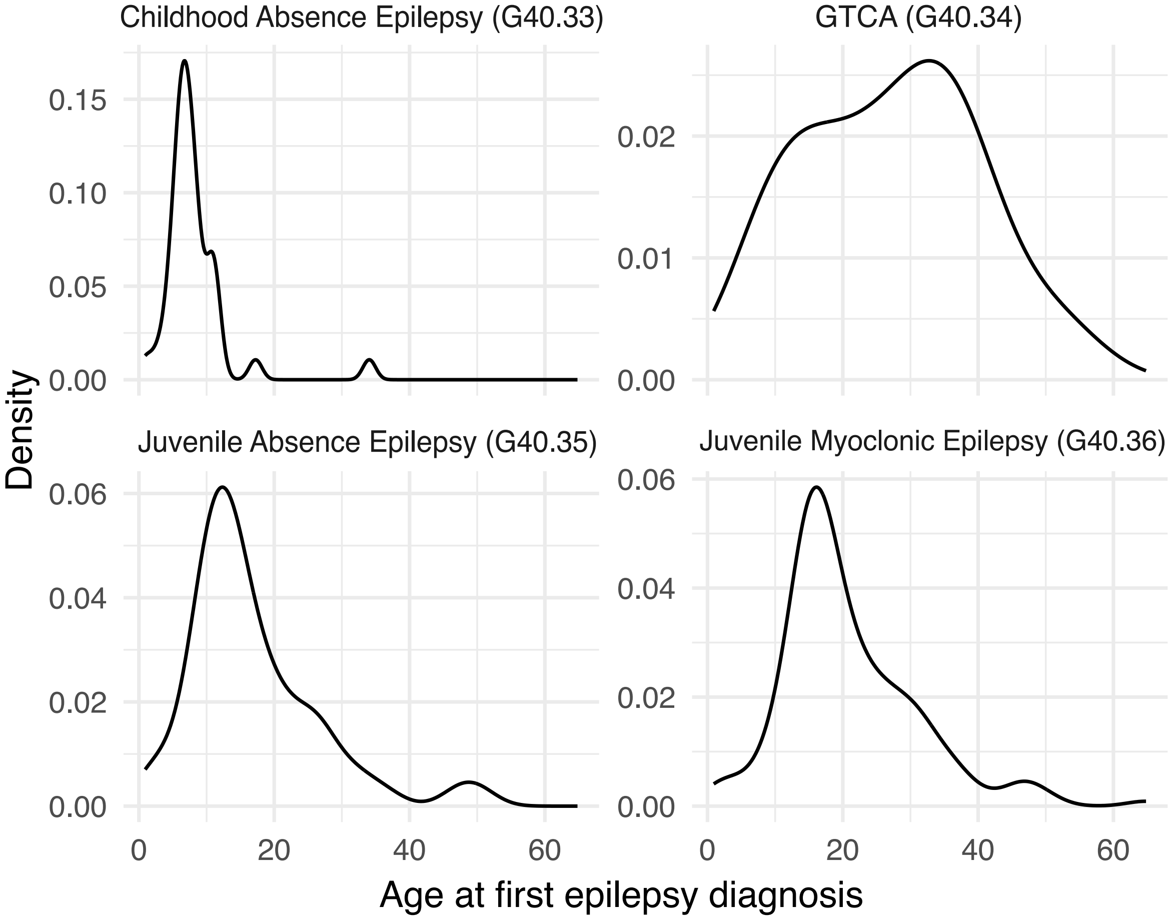

Supplementary Figure 3. **Epilepsy PRS as a marker for epilepsy risk across lifetime in 164,621 individuals from the Estonian biobank.** In panel A, we investigate the effect of PRS_{GGE} on GGE across lifetime; in panel B, we investigate the effect of PRS_{NAFE} on NAFE across lifetime. In each panel, on the left are density curves that display how samples are partitioned into six bins of PRS standard deviations. Survival curves in the middle give the cumulative epilepsy incidence (y-axis) across time (x-axis [years]) stratified for epilepsy PRS bins. The rightmost figures show epilepsy risk of each epilepsy PRS bin compared with the rest of the cohort (forest plots). Here, the point estimates represent hazard ratios (method: cox proportional hazards model), error bars show the 95%-confidence intervals.

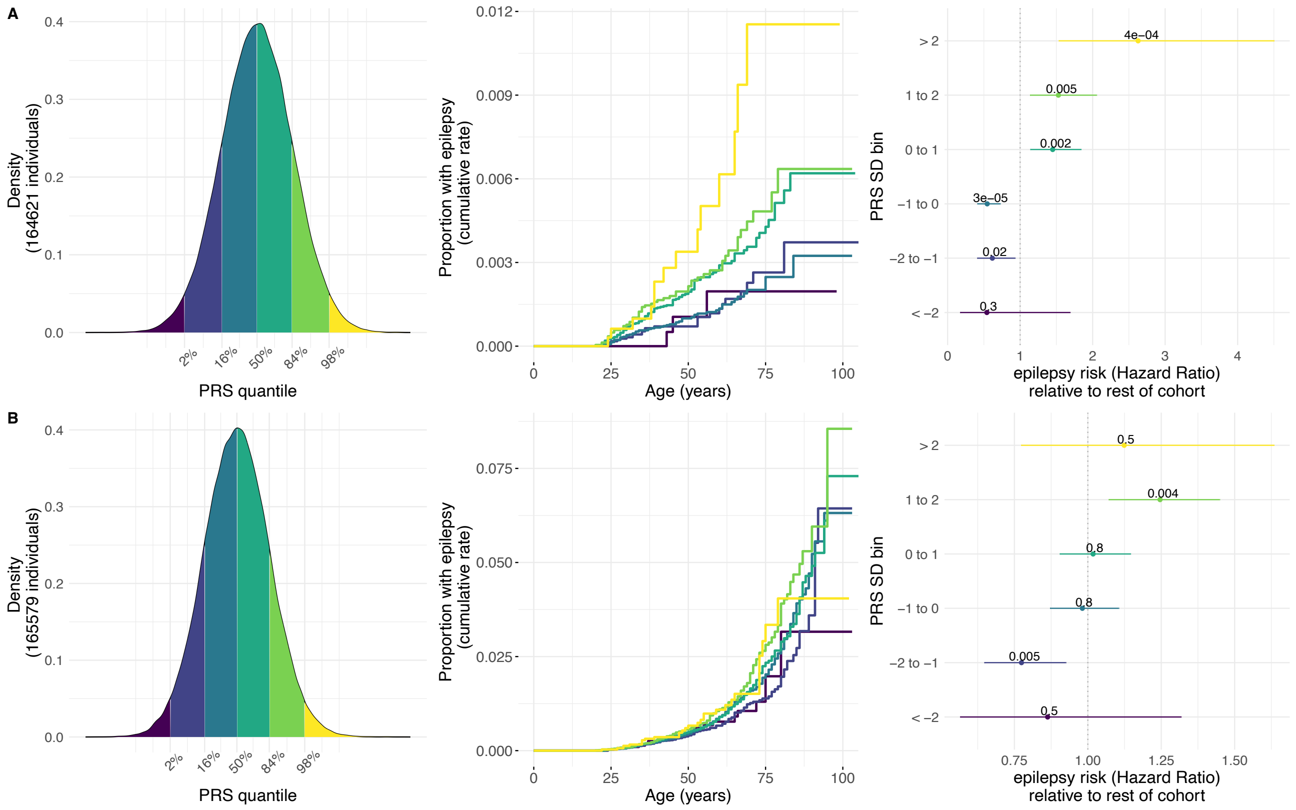

Supplementary Figure 4. Effect of epilepsy PRS_{GGE} on lifetime GGE incidence in 18,152 individuals with European (EUR), African (AFR) and American (AMR) ancestries from the Bio*Me* cohort (Mount Sinai Hospital, New York). In each panel, survival curves show the cumulative lifetime epilepsy incidence (y-axis) across age (x-axis [years]) stratified for bins of top (>75th percentile, blue), joined two middle (25th-75th percentile, green) and bottom quartiles (<25th percentile, red) of epilepsy PRS_{GGE}. Different panels show different genetic ancestry groups. A) EUR ancestry, B) AMR ancestry, C) AFR ancestry.

A

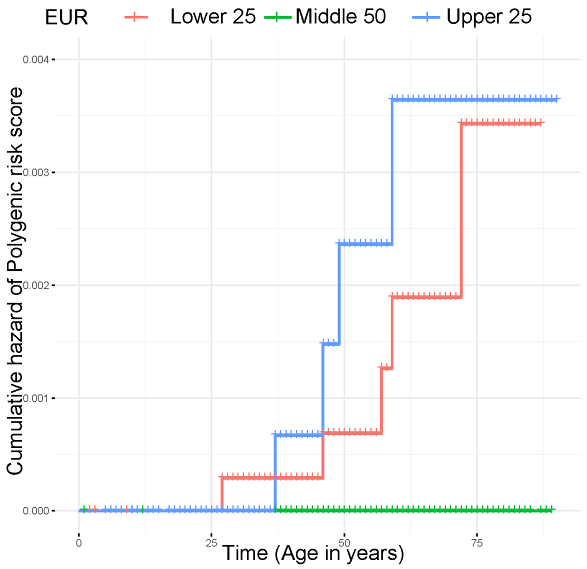

B

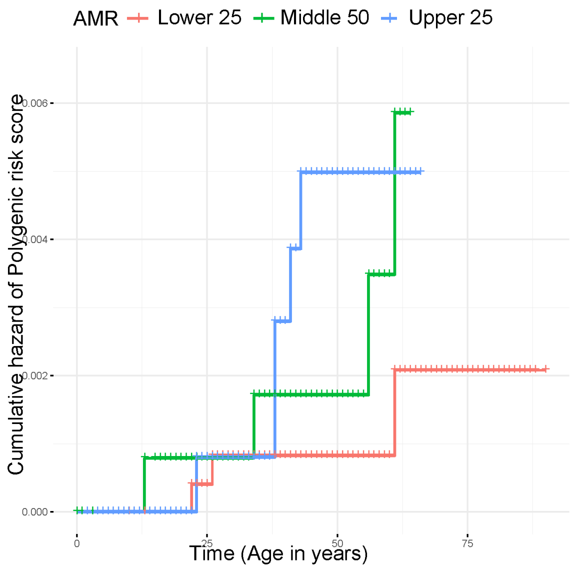

C

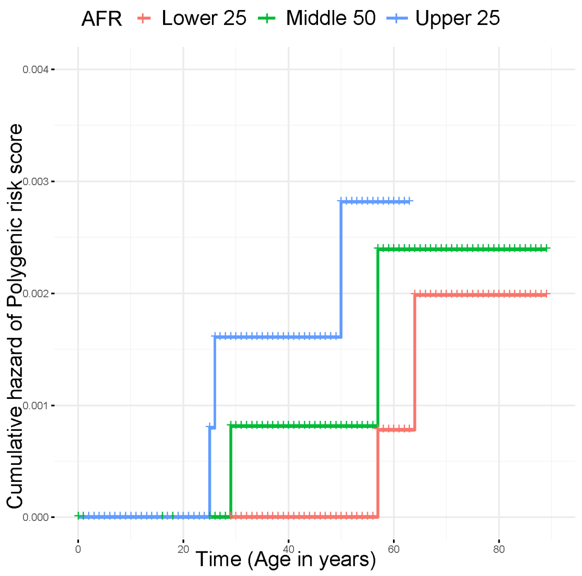

C

Supplementary Figure 5. **Sex-specific differences on the effect of PRS_{GGE} on epilepsy prevalence across lifetime.** Panels *Female* and *Male* indicate that the analysis was performed in only-female (n=257,496) and only-male (n=213,979) individuals, respectively. In both panels, on the left are density curves that display how samples are partitioned into six bins of PRS standard deviations. Survival curves in the middle give the cumulative epilepsy incidence (y-axis) across time (x-axis [years]) stratified for epilepsy PRS bins. The rightmost figures show epilepsy risk of each epilepsy PRS bin compared with the rest of the cohort (forest plots). Here, the point estimates represent hazard ratios (method: cox proportional hazards model), error bars show the 95%-confidence intervals.

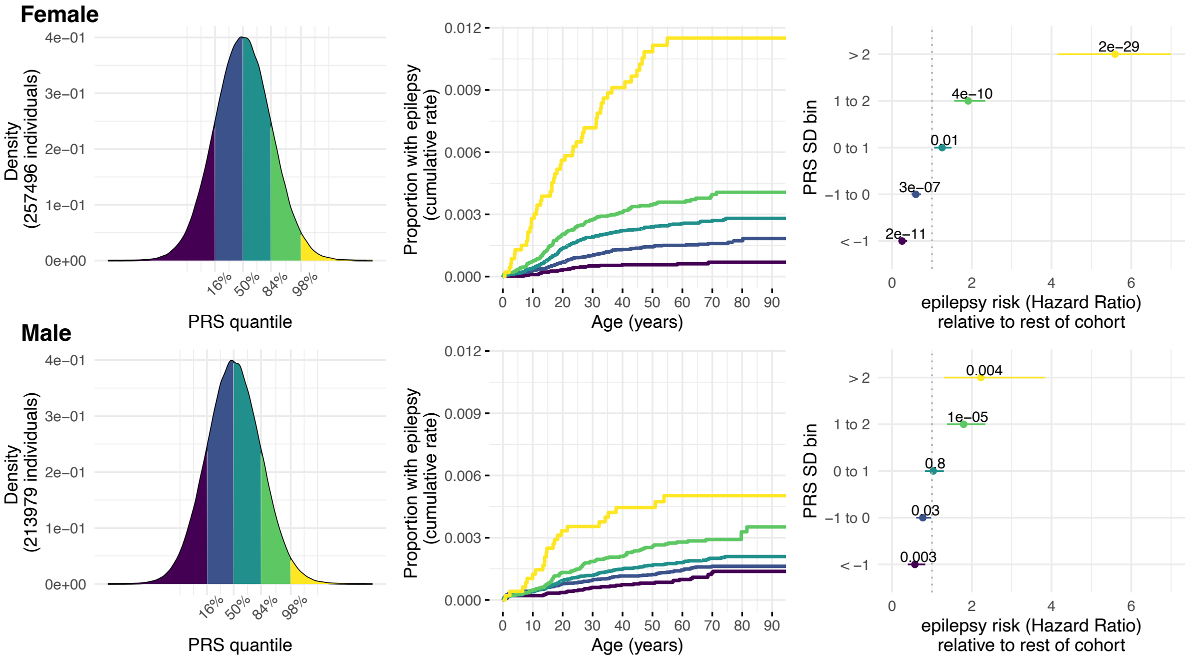

Supplementary Figure 6. **Sex-specific differences of age at first epilepsy diagnosis.** In all four panels are violin plots that display ages at first diagnoses for different subtypes of epilepsy, separately for males and females. Sample numbers are pasted at the top of the figure. Violin colors of red indicate female and grey indicate male. Sex was imputed from genotype data. Violin sizes are scaled to sample sizes.

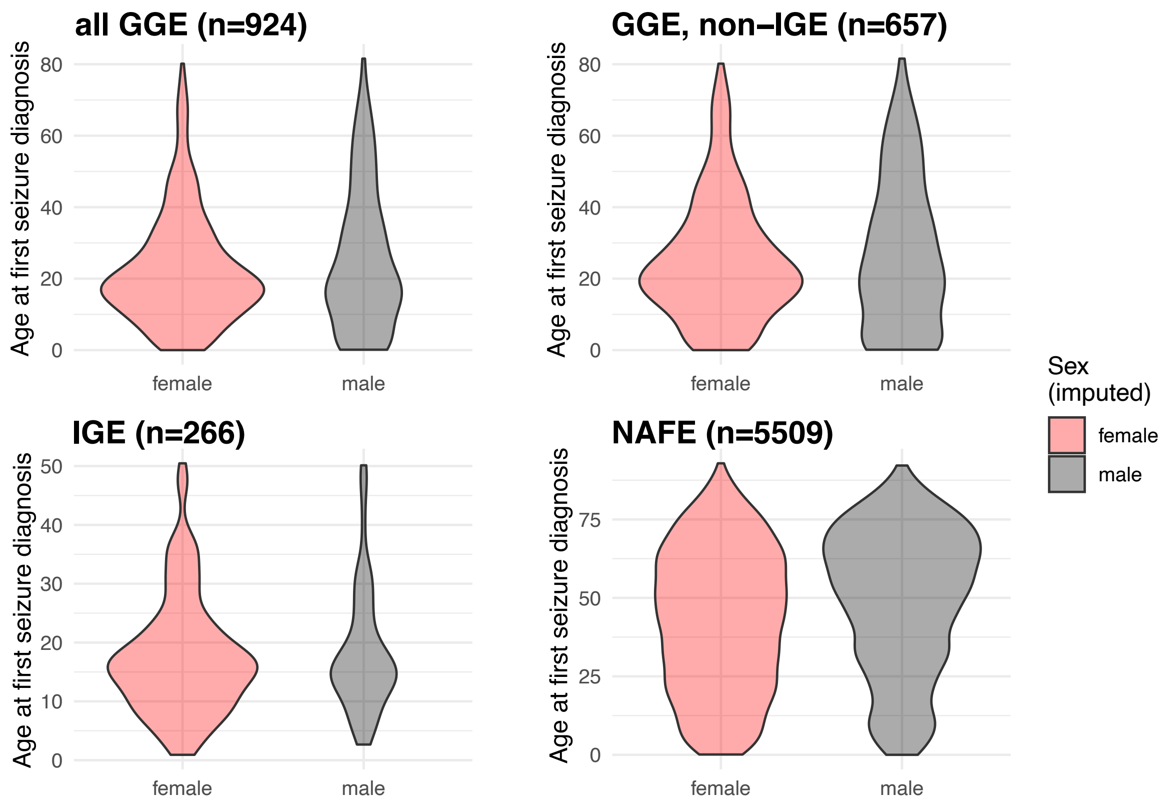

Supplementary Figure 7. Genetic correlations between NAFE and the 18 disease phenotypes that were significantly associated with PRS_{NAFE}. Disease phenotypes, sorted by clinical field, are shown on the x-axis. The genetic correlation coefficient (r_g) is visualized with a color scale ranging from -1 (blue) to 1 (red). Corresponding p-values of the correlations are shown inside the boxes. Genetic correlations were calculated with LD score regression [6]. The GWAS summary statistics were taken from FinnGen, release 10 (n = 430,897).

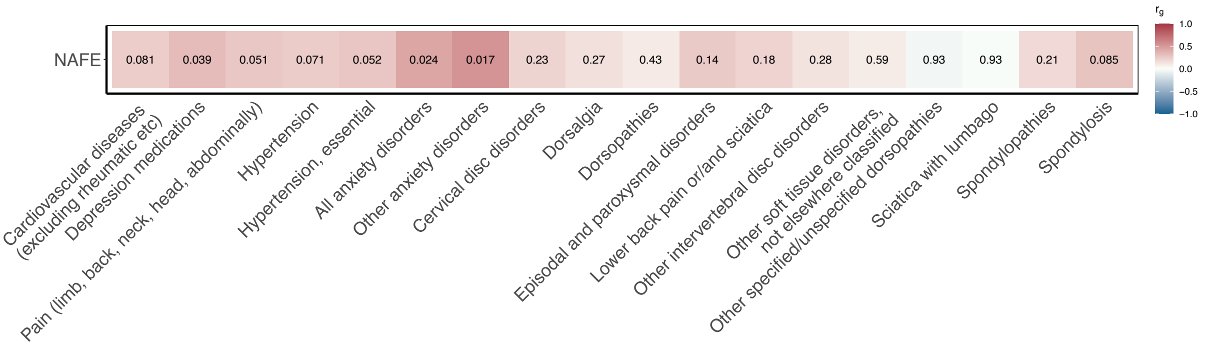

Supplementary Tables

Supplementary Table 1. Definitions of epilepsy cases and controls

Category	n<br>(FinnGen R12)	Definition
Control	470,551	Population controls that fulfilled none of the other definitions.
Focal	5,509	≥ 2x G40.0-G40.2;<br>≥ 2 ASM purchases<br>exclude: head injury, infection, tumor or stroke < 1 year before epilepsy (see Supplementary Table 2)
Generalized	924	≥ 2x G40.3;<br>≥ 2 ASM purchases
Other epilepsy diagnoses	5,077	≥ 1 x G40* or G41*
Single unspecified seizure	2,485	R56.8 or 7803A;<br>exclude: individuals with ASM purchase/ reimbursement within 10 years after and 2 years before seizure event; exclude individuals with head injury, infection, tumor or stroke < 1 year before seizure event
Possible alcohol withdrawal seizures	5,267	Multiple unspecified seizures/epilepsy OR<br>alcohol-related seizures OR<br>unspecified seizures/epilepsy & alcohol related diagnoses

Supplementary Table 2. Definitions of epilepsy ICD codes

Code	Category	ICD	Comment
**Unspecified seizure diagnosis codes**			
R56.8	unspecified	10	
7803A	unspecified	9	
**Focal and general epilepsy diagnosis codes**			
G40.3	generalized	10	
G40.30	generalized	10	
G40.31	generalized	10	
G40.33	generalized	10	IGE (Childhood Absence Epilepsy)
G40.34	generalized	10	IGE (Generalized Tonic–Clonic Seizures on Awakening)
G40.35	generalized	10	IGE (Juvenile absence epilepsy)
G40.36	generalized	10	IGE (Juvenile myoclonic epilepsy)
G40.39	generalized	10	
3450A	generalized	9	
3451A	generalized	9	
3451	generalized	9	
3452A	generalized	9	
3453A	generalized	9	
G40.0	focal	10	
G40.00	focal	10	
G40.01	focal	10	
G40.09	focal	10	
G40.1	focal	10	
G40.10	focal	10	
G40.11	focal	10	
G40.12	focal	10	
G40.19	focal	10	
G40.2	focal	10	
G40.20	focal	10	
G40.21	focal	10	
G40.22	focal	10	
G40.29	focal	10	
3454	focal	9	
3454A	focal	9	
3454B	focal	9	
3454X	focal	9	
3455A	focal	9	

Epilepsy syndromes, status epilepticus and other epilepsy diagnosis codes			
G41	unclassified epilepsy	10	Status epilepticus
G41.0	unclassified epilepsy	10	Status epilepticus
G41.1	unclassified epilepsy	10	Status epilepticus
G41.2	unclassified epilepsy	10	Status epilepticus
G41.8	unclassified epilepsy	10	Status epilepticus
G41.9	unclassified epilepsy	10	Status epilepticus
G40.50	unclassified epilepsy	10	Status epilepticus
3457A	unclassified epilepsy	9	Status epilepticus
3457B	unclassified epilepsy	9	Status epilepticus
3457X	unclassified epilepsy	9	Status epilepticus
G40	unclassified epilepsy	10	
G40.6	unclassified epilepsy	10	
G40.7	unclassified epilepsy	10	
G40.8	unclassified epilepsy	10	
G40.4	unclassified epilepsy	10	
G40.5	unclassified epilepsy	10	DEE
G40.59	unclassified epilepsy	10	DEE
G40.80	unclassified epilepsy	10	DEE
G40.89	unclassified epilepsy	10	DEE
G40.37	unclassified epilepsy	10	PME
345	unclassified	9	
3456A	unclassified epilepsy	9	DEE
3456B	unclassified epilepsy	9	GEFSplus
3458X	unclassified epilepsy	9	
G40.9	unclassified epilepsy	10	
3459X	unclassified epilepsy	9	
**Exclusion ICD codes**			
G40.51	alcohol related seizure	10	
C71	symptomatic focal epilepsy	10	Malignant neoplasm of brain
191	symptomatic focal epilepsy	9	Malignant neoplasm of brain
191	symptomatic focal epilepsy	8	Malignant neoplasm of brain
C70	symptomatic focal epilepsy	10	Malignant neoplasm of meninges
1921|1923	symptomatic focal epilepsy	9	Malignant neoplasm of meninges
192[1-2]	symptomatic focal epilepsy	8	Malignant neoplasm of meninges
C72	symptomatic focal epilepsy	10	Malignant neoplasm of spinal cord, cranial nerves and other parts of central nervous system
192	symptomatic focal epilepsy	9	Malignant neoplasm of spinal cord, cranial nerves and other parts of central nervous system
192	symptomatic focal epilepsy	8	Malignant neoplasm of spinal cord, cranial nerves and other parts of central nervous system

I6[0-4]|G45	symptomatic focal epilepsy	10	stroke
430|4330A|4331A|4339A|4340A|4341A|4349A|436|435	symptomatic focal epilepsy	9	stroke
430|431|433|434|435|436	symptomatic focal epilepsy	8	stroke
S00-S09	symptomatic focal epilepsy	10	head injury
G00-G09	symptomatic focal epilepsy	10	Inflammatory diseases of the central nervous system

Supplementary Table 3. Effect of $PRS_{all\text{-}epilepsy}$ on lifetime epilepsy risk.

Epilepsy risks are given as hazard ratios (HR) for epilepsy per SD increase of $PRS_{all\text{-}epilepsy}$. Method: Cox proportional hazards model. CI; confidence interval. Cohort: FinnGen.

PRS/ phenotype	OR	5%-CI	95%-CI	P-value	n cases	n controls
GGE	0.96	1.42	1.10	0.4	924	470,668
NAFE	1.12	1.09	1.16	1x10E-16	5,509	470,668
All epilepsy (≥2 epilepsy ICD codes	1.10	1.08	1.13	5x10E-19	9,313	470,668

Supplementary Table 4. Effect of epilepsy PRS on epilepsy at different ages of epilepsy onset.

Odds ratios for epilepsy case status were calculated comparing individuals without epilepsy to individuals with epilepsy age at seizure onset in respective age bins of 20 years. Epilepsy PRS was z-transformed, thus odds ratios are given per SD increase of epilepsy PRS. Method: logistic regression. Covariates: sex, birth year, age at last follow up, first 10 PCs of ancestry, genotyping batch. OR; Odds ratio, CI; confidence interval. Dataset: FinnGen.

PRS/ phenotype	age bin (years)	OR	5%-CI	95%-CI	P-value	n cases in bin	n controls in bin
GGE	0-20	1.90	1.72	2.09	9.17E-38	482	470551
GGE	20-40	1.66	1.47	1.88	1.20E-15	282	470551
GGE	40-60	1.70	1.40	2.07	1.49E-07	111	470551
GGE	60-80	1.09	0.80	1.47	0.59	46	470551
NAFE	0-20	1.19	1.11	1.27	4.73E-07	930	470551
NAFE	20-40	1.21	1.14	1.28	4.31E-10	1207	470551
NAFE	40-60	1.13	1.07	1.19	2.94E-06	1591	470551
NAFE	60-80	1.10	1.05	1.16	0.00017	1537	470551
NAFE	80-100	0.97	0.85	1.10	0.64	243	470551

Supplementary Notes

Replication in Bio*Me* cohort

Summary

We sought to replicate our analysis in other ancestry groups. We thus tested association of PRS_{GGE} with GGE in individuals with more diverse ancestries from the Bio*Me* cohort. Due to power, we restricted our analysis to populations of European (n= 7329), African (n= 5587) and American (n= 5236) genetic ancestry, respectively. In Supplementary Figure 5 we show survival curves stratifying the cumulative incidence of GGE according to polygenic risk quartiles across the respective genetic ancestries.

Methods

We replicated the analysis in the Bio*Me* cohort. PRS_{GGE} was calculated using summary statistics of the International League Against Epilepsy Consortium on Complex Epilepsies 2018 [2]. Epilepsy cases were derived from participants' EHR, using ICD-9 and -10 codes as described in Supplementary Table 2. Genetic ancestries of Bio*Me* participants were inferred using Principal Component Analysis (PCA) of genotypes using a random forest model. The model was trained using population labels from reference data of the 1000 genomes project, namely European, Admixed American, East Asian, South Asian, and African continental populations. Weight files for each continental ancestry group in the BioMe cohort were computed using the tool PRS-CS [3] and reference data of the 1000 genomes project, and PRSs of individuals calculated using PLINK [4]. The PRS_{GGE} was z-transformed to calculate HR per SD of PRS_{GGE} across lifetime with a Cox-proportional Hazards model.

Results

Epilepsy/PRS	N epilepsy	N total	Ancestry	HR per SD PRS_{GGE} (95%- CI)	p-value
GGE	10	7329	European	1.5 (0.8 - 2.8)	0.16
GGE	8	5587	American	1.0 (0.6 - 1.7)	0.95
GGE	14	5236	African	1.5 (0.7 - 3.1)	0.26
GGE	37	19255	European, African, American, South Asian, East Asian,	1.3 (0.9 - 1.7)	0.17

Effects of PRS on epilepsy risk in different cohorts. Epilepsy risks are given as hazard ratios (HR) for epilepsy per SD increase of epilepsy PRS. Method: Cox proportional hazards model. CI; confidence interval. Results are shown for ancestry groups with ≥ 3 epilepsy cases as well as a joint analysis of all individuals.

References

1 Hirsch, E. *et al.* ILAE definition of the Idiopathic Generalized Epilepsy Syndromes: Position statement by the ILAE Task Force on Nosology and Definitions. *Epilepsia* **63**, 1475-1499, doi:10.1111/epi.17236 (2022).

2 ILAE, Epilepsy, I. L. A. & Epilepsies), C. o. C. Genome-wide mega-analysis identifies 16 loci and highlights diverse biological mechanisms in the common epilepsies. *Nat Commun* **9**, 5269, doi:10.1038/s41467-018-07524-z (2018).

3 Ge, T., Chen, C. Y., Ni, Y., Feng, Y. A. & Smoller, J. W. Polygenic prediction via Bayesian regression and continuous shrinkage priors. *Nat Commun* **10**, 1776, doi:10.1038/s41467-019-09718-5 (2019).

4 Purcell, S. *et al.* PLINK: a tool set for whole-genome association and population-based linkage analyses. *Am J Hum Genet* **81**, 559-575, doi:10.1086/519795 (2007).

6 Bulik-Sullivan, B. et al. LD Score regression distinguishes confounding from polygenicity in genome-wide association studies. *Nat Genet **47**,* 291–295 (2015). https://doi.org/10.1038/ng.3211

FinnGen

Full Name	Affiliation	E-mail	Role 1	Role 2
Aarno Palotie	Institute for Molecular Medicine Finland (FIMM), HiLIFE, University of Helsinki,	aarno.palotie@helsinki.fi	Steering Committee	Steering Committee
Mark Daly	Institute for Molecular Medicine Finland (FIMM), HiLIFE, University of Helsinki,	mark.daly@helsinki.fi	Steering Committee	Steering Committee
Bridget Riley-Gills	Abbvie, Chicago, IL, United States	bridget.rileygillis@abbvie.com	Steering Committee	Pharmaceutical companies
Howard Jacob	Abbvie, Chicago, IL, United States	howard.jacob@abbvie.com	Steering Committee	Pharmaceutical companies
Coralie Viollet	Astra Zeneca, Cambridge, United Kingdom	coralie.viollet@astrazeneca.com	Steering Committee	Pharmaceutical companies
Slavé Petrovski	Astra Zeneca, Cambridge, United Kingdom	slav.petrovski@astrazeneca.com	Steering Committee	Pharmaceutical companies
Chia-Yen Chen	Biogen, Cambridge, MA, United States	chiayen.chen@biogen.com	Steering Committee	Pharmaceutical companies
Sally John	Biogen, Cambridge, MA, United States	sally.john@biogen.com	Steering Committee	Pharmaceutical companies
George Okafo	Boehringer Ingelheim, Ingelheim am Rhein, Germany	george.okafo@boehringer-ingelheim.com	Steering Committee	Pharmaceutical companies
Robert Plenge	Bristol Myers Squibb, New York, NY, United States	robert.plenge@bms.com	Steering Committee	Pharmaceutical companies
Joseph Maranville	Bristol Myers Squibb, New York, NY, United States	joseph.maranville@bms.com	Steering Committee	Pharmaceutical companies
Mark McCarthy	Genentech, San Francisco, CA, United States	mccarthy.mark@gene.com	Steering Committee	Pharmaceutical companies
Rion Pendergrass	Genentech, San Francisco, CA, United States	penders2@gene.com	Steering Committee	Pharmaceutical companies
Margaret G. Ehm	GlaxoSmithKline, Collegeville, PA, United States	meg.g.ehm@gsk.com	Steering Committee	Pharmaceutical companies
Kirsi Auro	GlaxoSmithKline, Espoo, Finland	kirsi.m.auro@gsk.com	Steering Committee	Pharmaceutical companies
Simonne Longerich	Merck, Kenilworth, NJ, United States	simonne.longerich@merck.com	Steering Committee	Pharmaceutical companies
Anders Mälarstig	Pfizer, New York, NY, United States	anders.malarstig@pfizer.com	Steering Committee	Pharmaceutical companies
Anna Vlahiotis	Pfizer, New York, NY, United States	anna.vlahiotis@pfizer.com	Steering Committee	Pharmaceutical companies
Katherine Klinger	Translational Sciences, Sanofi R&D, Framingham, MA, USA	katherine.klinger@sanofi.com	Steering Committee	Pharmaceutical companies
Clement Chatelain	Translational Sciences, Sanofi R&D, Framingham, MA, USA	clement.chatelain@sanofi.com	Steering Committee	Pharmaceutical companies
Matthias Gossel	Translational Sciences, Sanofi R&D, Framingham, MA, USA	matthias.gossel@sanofi.com	Steering Committee	Pharmaceutical companies
Karol Estrada	Maze Therapeutics, San Francisco, CA, United States	kestrada@mazetx.com	Steering Committee	Pharmaceutical companies
Robert Graham	Maze Therapeutics, San Francisco, CA, United States	rgraham@mazetx.com	Steering Committee	Pharmaceutical companies
Dawn Waterworth	Janssen Research & Development, LLC, Spring House, PA, United States	dwaterwo@its.jnj.com	Steering Committee	Pharmaceutical companies
Chris O´Donnell	Novartis Institutes for BioMedical Research, Cambridge, MA, United States	chris.odonnell@novartis.com	Steering Committee	Pharmaceutical companies
Nicole Renaud	Novartis Institutes for BioMedical Research, Cambridge, MA, United States	nicole.renaud@novartis.com	Steering Committee	Pharmaceutical companies
Tomi P. Mäkelä	HiLIFE, University of Helsinki, Finland, Finland	tomi.makela@helsinki.fi	Steering Committee	University of Helsinki & Biobanks
Jaakko Kaprio	Institute for Molecular Medicine Finland (FIMM), HiLIFE, University of Helsinki,	jaakko.kaprio@helsinki.fi	Steering Committee	University of Helsinki & Biobanks
Minna Ruddock	Arctic biobank / University of Oulu	minna.ruddock@oulu.fi	Steering Committee	University of Helsinki & Biobanks
Petri Virolainen	Auria Biobank / University of Turku / Hospital District of Southwest Finland, Turku,	petri.virolainen@tyks.fi	Steering Committee	University of Helsinki & Biobanks
Antti Hakanen	Auria Biobank / University of Turku / Hospital District of Southwest Finland, Turku,	antti.hakanen@tyks.fi	Steering Committee	University of Helsinki & Biobanks
Terhi Kilpi	THL Biobank / Finnish Institute for Health and Welfare (THL), Helsinki, Finland	terhi.kilpi@thl.fi	Steering Committee	University of Helsinki & Biobanks
Markus Perola	THL Biobank / Finnish Institute for Health and Welfare (THL), Helsinki, Finland	markus.perola@thl.fi	Steering Committee	University of Helsinki & Biobanks
Jukka Partanen	Finnish Red Cross Blood Service / Finnish Hematology Registry and Clinical	jukka.partanen@veripalvelu.fi	Steering Committee	University of Helsinki & Biobanks
Taneli Raivio	Helsinki Biobank / Helsinki University and Hospital District of Helsinki and Uusimaa,	taneli.raivio@hus.fi	Steering Committee	University of Helsinki & Biobanks
Jani Tikkanen	Northern Finland Biobank Borealis / University of Oulu / Northern Ostrobothnia	jani.tikkanen@ppshp.fi	Steering Committee	University of Helsinki & Biobanks
Raisa Serpi	Northern Finland Biobank Borealis / University of Oulu / Northern Ostrobothnia	raisa.serpi@ppshp.fi	Steering Committee	University of Helsinki & Biobanks
Kati Kristiansson	Finnish Clinical Biobank Tampere / University of Tampere / Pirkanmaa Hospital	kati.kristiansson@pirha.fi	Steering Committee	University of Helsinki & Biobanks
Veli-Matti Kosma	Biobank of Eastern Finland / University of Eastern Finland / Northern Savo Hospital	veli-matti.kosma@uef.fi	Steering Committee	University of Helsinki & Biobanks
Jari Laukkanen	Central Finland Biobank / University of Jyväskylä / Central Finland Health Care	jari.laukkanen@ksshp.fi	Steering Committee	University of Helsinki & Biobanks
Marco Hautalahti	FINBB - Finnish biobank cooperative	marco.hautalahti@finbb.fi	Steering Committee	University of Helsinki & Biobanks
Outi Tuovila	Business Finland, Helsinki, Finland	outi.tuovila@businessfinland.fi	Steering Committee	Other Experts/ Non-Voting Members
Jeffrey Waring	Abbvie, Chicago, IL, United States	jeff.waring@abbvie.com	Scientific Committee	Pharmaceutical companies
Bridget Riley-Gillis	Abbvie, Chicago, IL, United States	bridget.rileygillis@abbvie.com	Scientific Committee	Pharmaceutical companies
Fedik Rahimov	Abbvie, Chicago, IL, United States	fedik.rahimov@abbvie.com	Scientific Committee	Pharmaceutical companies
Ioanna Tachmazidou	Astra Zeneca, Cambridge, United Kingdom	ioanna.tachmazidou@astrazeneca.com	Scientific Committee	Pharmaceutical companies
Chia-Yen Chen	Biogen, Cambridge, MA, United States	chiayen.chen@biogen.com	Scientific Committee	Pharmaceutical companies
Zhihao Ding	Boehringer Ingelheim, Ingelheim am Rhein, Germany	zhihao.ding@boehringer-ingelheim.com	Scientific Committee	Pharmaceutical companies
Marc Jung	Boehringer Ingelheim, Ingelheim am Rhein, Germany	marc_oliver.jung@boehringer-ingelheim.c	Scientific Committee	Pharmaceutical companies
Hanati Tuoken	Boehringer Ingelheim, Ingelheim am Rhein, Germany	hanati.tuoken@boehringer-ingelheim.com	Scientific Committee	Pharmaceutical companies
Shameek Biswas	Bristol Myers Squibb, New York, NY, United States	Shameek.Biswas@bms.com	Scientific Committee	Pharmaceutical companies
Rion Pendergrass	Genentech, San Francisco, CA, United States	penders2@gene.com	Scientific Committee	Pharmaceutical companies
Margaret G. Ehm	GlaxoSmithKline, Collegeville, PA, United States	meg.g.ehm@gsk.com	Scientific Committee	Pharmaceutical companies
David Pulford	GlaxoSmithKline, Stevenage, United Kingdom	david.x.pulford@gsk.com	Scientific Committee	Pharmaceutical companies
Neha Raghavan	Merck, Kenilworth, NJ, United States	neha.raghavan@merck.com	Scientific Committee	Pharmaceutical companies
Adriana Huertas-Vazque	Merck, Kenilworth, NJ, United States	adriana.huertas.vazquez@merck.com	Scientific Committee	Pharmaceutical companies
Jae-Hoon Sul	Merck, Kenilworth, NJ, United States	jae.hoon.sul@merck.com	Scientific Committee	Pharmaceutical companies
Anders Mälarstig	Pfizer, New York, NY, United States	anders.malarstig@pfizer.com	Scientific Committee	Pharmaceutical companies
Xinli Hu	Pfizer, New York, NY, United States	xinli.hu@pfizer.com	Scientific Committee	Pharmaceutical companies
Åsa Hedman	Pfizer, New York, NY, United States	asa.hedman@pfizer.com	Scientific Committee	Pharmaceutical companies
Katherine Klinger	Translational Sciences, Sanofi R&D, Framingham, MA, USA	katherine.klinger@sanofi.com	Scientific Committee	Pharmaceutical companies
Robert Graham	Maze Therapeutics, San Francisco, CA, United States	rgraham@mazetx.com	Scientific Committee	Pharmaceutical companies
Dawn Waterworth	Janssen Research & Development, LLC, Spring House, PA, United States	dwaterwo@its.jnj.com	Scientific Committee	Pharmaceutical companies
Nicole Renaud	Novartis Institutes for BioMedical Research, Cambridge, MA, United States	nicole.renaud@novartis.com	Scientific Committee	Pharmaceutical companies
Ma´en Obeidat	Novartis Institutes for BioMedical Research, Cambridge, MA, United States	maen.obeidat@novartis.com	Scientific Committee	Pharmaceutical companies
Jonathan Chung	Novartis Institutes for BioMedical Research, Cambridge, MA, United States	jonathan.chung@novartis.com	Scientific Committee	Pharmaceutical companies
Jonas Zierer	Novartis Institutes for BioMedical Research, Cambridge, MA, United States	jonas.zierer@novartis.com	Scientific Committee	Pharmaceutical companies
Mari Niemi	Novartis Institutes for BioMedical Research, Cambridge, MA, United States	mari.niemi@novartis.com	Scientific Committee	Pharmaceutical companies
Samuli Ripatti	Institute for Molecular Medicine Finland (FIMM), HiLIFE, University of Helsinki, Helsink	samuli.ripatti@helsinki.fi	Scientific Committee	University of Helsinki & Biobanks
Johanna Schleutker	Auria Biobank / Univ. of Turku / Hospital District of Southwest Finland, Turku, Finland	johanna.schleutker@utu.fi	Scientific Committee	University of Helsinki & Biobanks
Markus Perola	THL Biobank / Finnish Institute for Health and Welfare (THL), Helsinki, Finland	markus.perola@thl.fi	Scientific Committee	University of Helsinki & Biobanks
Mikko Arvas	Finnish Red Cross Blood Service / Finnish Hematology Registry and Clinical Biobank, Helsinki, Finland	mikko.arvas@veripalvelu.fi	Scientific Committee	University of Helsinki & Biobanks
Olli Carpén	Helsinki Biobank / Helsinki University and Hospital District of Helsinki and Uusimaa, Helsinki	olli.carpen@helsinki.fi	Scientific Committee	University of Helsinki & Biobanks
Reetta Hinttala	Northern Finland Biobank Borealis / University of Oulu / Northern Ostrobothnia Hospital District, Oulu, Finland	reetta.hinttala@oulu.fi	Scientific Committee	University of Helsinki & Biobanks
Johannes Kettunen	Northern Finland Biobank Borealis / University of Oulu / Northern Ostrobothnia Hospital District, Oulu, Finland	johannes.kettunen@oulu.fi	Scientific Committee	University of Helsinki & Biobanks
Arto Mannermaa	Biobank of Eastern Finland / University of Eastern Finland / Northern Savo Hospital District, Kuopio, Finland	arto.mannermaa@uef.fi	Scientific Committee	University of Helsinki & Biobanks
Katriina Aalto-Setälä	Faculty of Medicine and Health Technology, Tampere University, Tampere, Finland	katriina.aalto-setala@tuni.fi	Scientific Committee	University of Helsinki & Biobanks
Mika Kähönen	Finnish Clinical Biobank Tampere / University of Tampere / Pirkanmaa Hospital District, Tampere, Finland	mika.kahonen@uta.fi	Scientific Committee	University of Helsinki & Biobanks
Jari Laukkanen	Central Finland Biobank / University of Jyväskylä / Central Finland Health Care District, Jyväskylä, Finland	jari.laukkanen@ksshp.fi	Scientific Committee	University of Helsinki & Biobanks
Johanna Mäkelä	FINBB - Finnish biobank cooperative	johanna.makela@finbb.fi	Scientific Committee	University of Helsinki & Biobanks
Reetta Kälviäinen	Northern Savo Hospital District, Kuopio, Finland	reetta.kalviainen@kuh.fi	Clinical Groups	Neurology Group
Valtteri Julkunen	Northern Savo Hospital District, Kuopio, Finland	valtteri.julkunen@kuh.fi	Clinical Groups	Neurology Group
Hilkka Soininen	Northern Savo Hospital District, Kuopio, Finland	hilkka.soininen@uef.fi	Clinical Groups	Neurology Group
Anne Remes	Northern Ostrobothnia Hospital District, Oulu, Finland	anne.remes@oulu.fi	Clinical Groups	Neurology Group
Mikko Hiltunen	University of Eastern Finland, Kuopio, Finland	mikko.hiltunen@uef.fi	Clinical Groups	Neurology Group
Jukka Peltola	Pirkanmaa Hospital District, Tampere, Finland	jukka.peltola@pshp.fi	Clinical Groups	Neurology Group
Minna Raivio	Hospital District of Helsinki and Uusimaa, Helsinki, Finland	minna.raivio@geri.fi	Clinical Groups	Neurology Group
Pentti Tienari	Hospital District of Helsinki and Uusimaa, Helsinki, Finland	pentti.tienari@hus.fi	Clinical Groups	Neurology Group
Juha Rinne	Hospital District of Southwest Finland, Turku, Finland	juha.rinne@tyks.fi	Clinical Groups	Neurology Group
Roosa Kallionpää	Hospital District of Southwest Finland, Turku, Finland	roosa.kallionpaa@tyks.fi	Clinical Groups	Neurology Group
Juulia Partanen	Institute for Molecular Medicine Finland, HiLIFE, University of Helsinki, Finland	juulia.partanen@helsinki.fi	Clinical Groups	Neurology Group
Adam Ziemann	Abbvie, Chicago, IL, United States	adam.ziemann@abbvie.com	Clinical Groups	Neurology Group
Nizar Smaoui	Abbvie, Chicago, IL, United States	nizar.smaoui@abbvie.com	Clinical Groups	Neurology Group
Anne Lehtonen	Abbvie, Chicago, IL, United States	anne.lehtonen@abbvie.com	Clinical Groups	Neurology Group
Susan Eaton	Biogen, Cambridge, MA, United States	susan.eaton@biogen.com	Clinical Groups	Neurology Group
Heiko Runz	Biogen, Cambridge, MA, United States	heiko.runz@biogen.com	Clinical Groups	Neurology Group
Sanni Lahdenperä	Biogen, Cambridge, MA, United States	sanni.lahdenpera@biogen.com	Clinical Groups	Neurology Group
Shameek Biswas	Bristol Myers Squibb, New York, NY, United States	shameek.biswas@bms.com	Clinical Groups	Neurology Group
Natalie Bowers	Genentech, San Francisco, CA, United States	bowersn1@gene.com	Clinical Groups	Neurology Group
Edmond Teng	Genentech, San Francisco, CA, United States	teng.edmond@gene.com	Clinical Groups	Neurology Group
Rion Pendergrass	Genentech, San Francisco, CA, United States	penders2@gene.com	Clinical Groups	Neurology Group
Fanli Xu	GlaxoSmithKline, Brentford, United Kingdom	chun-fang.2.xu@gsk.com	Clinical Groups	Neurology Group
David Pulford	GlaxoSmithKline, Stevenage, United Kingdom	david.x.pulford@gsk.com	Clinical Groups	Neurology Group
Kirsi Auro	GlaxoSmithKline, Espoo, Finland	kirsi.m.auro@gsk.com	Clinical Groups	Neurology Group

Laura Addis	GlaxoSmithKline, Brentford, United Kingdom	laura.x.addis@gsk.com	Clinical Groups	**Neurology Group**
John Eicher	GlaxoSmithKline, Brentford, United Kingdom	john.d.eicher@gsk.com	Clinical Groups	**Neurology Group**
Qingqin S Li	Janssen Research & Development, LLC, Titusville, NJ 08560, United States	QLi2@its.jnj.com	Clinical Groups	**Neurology Group**
Karen He	Janssen Research & Development, LLC, Spring House, PA, United States	khe2@its.jnj.com	Clinical Groups	**Neurology Group**
Ekaterina Khramtsova	Janssen Research & Development, LLC, Spring House, PA, United States	ekhramts@its.jnj.com	Clinical Groups	**Neurology Group**
Neha Raghavan	Merck, Kenilworth, NJ, United States	neha.raghavan@merck.com	Clinical Groups	**Neurology Group**
Martti Färkkilä	Hospital District of Helsinki and Uusimaa, Helsinki, Finland	martti.farkkila@hus.fi	Clinical Groups	**Gastroenterology Group**
Jukka Koskela	Hospital District of Helsinki and Uusimaa, Helsinki, Finland	jukka.koskela@helsinki.fi	Clinical Groups	**Gastroenterology Group**
Sampsa Pikkarainen	Hospital District of Helsinki and Uusimaa, Helsinki, Finland	sampsa.pikkarainen@hus.fi	Clinical Groups	**Gastroenterology Group**
Airi Jussila	Pirkanmaa Hospital District, Tampere, Finland	airi.jussila@pshp.fi	Clinical Groups	**Gastroenterology Group**
Katri Kaukinen	Pirkanmaa Hospital District, Tampere, Finland	katri.kaukinen@tuni.fi	Clinical Groups	**Gastroenterology Group**
Timo Blomster	Northern Ostrobothnia Hospital District, Oulu, Finland	timo.blomster@ppshp.fi	Clinical Groups	**Gastroenterology Group**
Mikko Kiviniemi	Northern Savo Hospital District, Kuopio, Finland	mikko.kiviniemi@kuh.fi	Clinical Groups	**Gastroenterology Group**
Markku Voutilainen	Hospital District of Southwest Finland, Turku, Finland	markku.voutilainen@tyks.fi	Clinical Groups	**Gastroenterology Group**
Mark Daly	Institute for Molecular Medicine, Finland (FIMM), HiLIFE, University of Helsinki, Helsinki, Finland; Broad Institute of MIT and Harvard; Massachusetts General Hospital	mark.daly@helsinki.fi	Clinical Groups	**Gastroenterology Group**
Jeffrey Waring	Abbvie, Chicago, IL, United States	jeff.waring@abbvie.com	Clinical Groups	**Gastroenterology Group**
Nizar Smaoui	Abbvie, Chicago, IL, United States	nizar.smaoui@abbvie.com	Clinical Groups	**Gastroenterology Group**
Fedik Rahimov	Abbvie, Chicago, IL, United States	fedik.rahimov@abbvie.com	Clinical Groups	**Gastroenterology Group**
Anne Lehtonen	Abbvie, Chicago, IL, United States	anne.lehtonen@abbvie.com	Clinical Groups	**Gastroenterology Group**
Tim Lu	Genentech, San Francisco, CA, United States	lut8@gene.com	Clinical Groups	**Gastroenterology Group**
Natalie Bowers	Genentech, San Francisco, CA, United States	bowersn1@gene.com	Clinical Groups	**Gastroenterology Group**
Rion Pendergrass	Genentech, San Francisco, CA, United States	penders2@gene.com	Clinical Groups	**Gastroenterology Group**
Linda McCarthy	GlaxoSmithKline, Brentford, United Kingdom	linda.c.mccarthy@gsk.com	Clinical Groups	**Gastroenterology Group**
Amy Hart	Janssen Research & Development, LLC, Spring House, PA, United States	ahart13@its.jnj.com	Clinical Groups	**Gastroenterology Group**
Meijian Guan	Janssen Research & Development, LLC, Spring House, PA, United States	mguan4@its.jnj.com	Clinical Groups	**Gastroenterology Group**
Jason Miller	Merck, Kenilworth, NJ, United States	jason.miller4@merck.com	Clinical Groups	**Gastroenterology Group**
Kirsi Kalpala	Pfizer, New York, NY, United States	kirsi.kalpala@pfizer.com	Clinical Groups	**Gastroenterology Group**
Melissa Miller	Pfizer, New York, NY, United States	melissa.r.miller@pfizer.com	Clinical Groups	**Gastroenterology Group**
Xinli Hu	Pfizer, New York, NY, United States	xinli.hu@pfizer.com	Clinical Groups	**Gastroenterology Group**
Kari Eklund	Hospital District of Helsinki and Uusimaa, Helsinki, Finland	kari.eklund@hus.fi	Clinical Groups	**Rheumatology Group**
Antti Palomäki	Hospital District of Southwest Finland, Turku, Finland	ajpalo@utu.fi	Clinical Groups	**Rheumatology Group**
Pia Isomäki	Pirkanmaa Hospital District, Tampere, Finland	pia.isomaki@pshp.fi	Clinical Groups	**Rheumatology Group**
Laura Pirilä	Hospital District of Southwest Finland, Turku, Finland	laura.pirila@fimnet.fi,laura.pirila@tyks.fi	Clinical Groups	**Rheumatology Group**
Oili Kaipiainen-Seppäne	Northern Savo Hospital District, Kuopio, Finland	oili.kaipiainen-seppanen@kuh.fi	Clinical Groups	**Rheumatology Group**
Johanna Huhtakangas	Northern Ostrobothnia Hospital District, Oulu, Finland	johanna.huhtakangas@kuh.fi	Clinical Groups	**Rheumatology Group**
Nina Mars	Institute for Molecular Medicine Finland (FIMM), HiLIFE, University of Helsinki, Helsink	nina.mars@helsinki.fi	Clinical Groups	**Rheumatology Group**
Jeffrey Waring	Abbvie, Chicago, IL, United States	jeff.waring@abbvie.com	Clinical Groups	**Rheumatology Group**
Fedik Rahimov	Abbvie, Chicago, IL, United States	fedik.rahimov@abbvie.com	Clinical Groups	**Rheumatology Group**
Apinya Lertratanakul	Abbvie, Chicago, IL, United States	apinya.lertratanakul@abbvie.com	Clinical Groups	**Rheumatology Group**
Nizar Smaoui	Abbvie, Chicago, IL, United States	nizar.smaoui@abbvie.com	Clinical Groups	**Rheumatology Group**
Anne Lehtonen	Abbvie, Chicago, IL, United States	anne.lehtonen@abbvie.com	Clinical Groups	**Rheumatology Group**
Coralie Viollet	AstraZeneca, Cambridge, United Kingdom	coralie.viollet@astrazeneca.com	Clinical Groups	**Rheumatology Group**
Marla Hochfeld	Bristol Myers Squibb, New York, NY, United States	mhochfeld@celgene.com	Clinical Groups	**Rheumatology Group**
Natalie Bowers	Genentech, San Francisco, CA, United States	bowersn1@gene.com	Clinical Groups	**Rheumatology Group**
Rion Pendergrass	Genentech, San Francisco, CA, United States	penders2@gene.com	Clinical Groups	**Rheumatology Group**
Jorge Esparza Gordillo	GlaxoSmithKline, Brentford, United Kingdom	jorge.x.esparza-gordillo@gsk.com	Clinical Groups	**Rheumatology Group**
Kirsi Auro	GlaxoSmithKline, Espoo, Finland	kirsi.m.auro@gsk.com	Clinical Groups	**Rheumatology Group**
Dawn Waterworth	Janssen Research & Development, LLC, Spring House, PA, United States	dwaterwo@its.jnj.com	Clinical Groups	**Rheumatology Group**
Fabiana Farias	Merck, Kenilworth, NJ, United States	fabiana.farias@merck.com	Clinical Groups	**Rheumatology Group**
Kirsi Kalpala	Pfizer, New York, NY, United States	kirsi.kalpala@pfizer.com	Clinical Groups	**Rheumatology Group**
Nan Bing	Pfizer, New York, NY, United States	nan.bing@pfizer.com	Clinical Groups	**Rheumatology Group**
Xinli Hu	Pfizer, New York, NY, United States	xinli.hu@pfizer.com	Clinical Groups	**Rheumatology Group**
Tarja Laitinen	Pirkanmaa Hospital District, Tampere, Finland	tarja.laitinen@pshp.fi	Clinical Groups	**Pulmonology Group**
Margit Pelkonen	Northern Savo Hospital District, Kuopio, Finland	margit.pelkonen@kuh.fi	Clinical Groups	**Pulmonology Group**
Paula Kauppi	Hospital District of Helsinki and Uusimaa, Helsinki, Finland	paula.kauppi@hus.fi	Clinical Groups	**Pulmonology Group**
Hannu Kankaanranta	University of Gothenburg, Gothenburg, Sweden/ Seinäjoki Central Hospital, Seinäjoki, Finland/ Tampere University, Tampere, Finland	hannu.kankaanranta@tuni.fi	Clinical Groups	**Pulmonology Group**
Terttu Harju	Northern Ostrobothnia Hospital District, Oulu, Finland	terttu.harju@oulu.fi	Clinical Groups	**Pulmonology Group**
Riitta Lahesmaa	Hospital District of Southwest Finland, Turku, Finland	rilahes@utu.fi	Clinical Groups	**Pulmonology Group**
Nizar Smaoui	Abbvie, Chicago, IL, United States	nizar.smaoui@abbvie.com	Clinical Groups	**Pulmonology Group**
Coralie Viollet	AstraZeneca, Cambridge, United Kingdom	coralie.viollet@astrazeneca.com	Clinical Groups	**Pulmonology Group**
Susan Eaton	Biogen, Cambridge, MA, United States	susan.eaton@biogen.com	Clinical Groups	**Pulmonology Group**
Hubert Chen	Genentech, San Francisco, CA, United States	chenh37@gene.com	Clinical Groups	**Pulmonology Group**
Rion Pendergrass	Genentech, San Francisco, CA, United States	penders2@gene.com	Clinical Groups	**Pulmonology Group**
Natalie Bowers	Genentech, San Francisco, CA, United States	bowersn1@gene.com	Clinical Groups	**Pulmonology Group**
Joanna Betts	GlaxoSmithKline, Brentford, United Kingdom	joanna.c.betts@gsk.com	Clinical Groups	**Pulmonology Group**
Kirsi Auro	GlaxoSmithKline, Espoo, Finland	kirsi.m.auro@gsk.com	Clinical Groups	**Pulmonology Group**
Rajashree Mishra	GlaxoSmithKline, Brentford, United Kingdom	rajashree.x.mishra@gsk.com	Clinical Groups	**Pulmonology Group**
Majd Mouded	Novartis, Basel, Switzerland	majd.mouded@novartis.com	Clinical Groups	**Pulmonology Group**
Debby Ngo	Novartis, Basel, Switzerland	debby.ngo@novartis.com	Clinical Groups	**Pulmonology Group**
Teemu Niiranen	Finnish Institute for Health and Welfare (THL), Helsinki, Finland	teemu.niiranen@thl.fi	Clinical Groups	**Cardiometabolic Diseases Group**
Felix Vaura	Finnish Institute for Health and Welfare (THL), Helsinki, Finland	fechva@utu.fi	Clinical Groups	**Cardiometabolic Diseases Group**
Veikko Salomaa	Finnish Institute for Health and Welfare (THL), Helsinki, Finland	veikko.salomaa@thl.fi	Clinical Groups	**Cardiometabolic Diseases Group**
Kaj Metsärinne	Hospital District of Southwest Finland, Turku, Finland	kaj.metsarinne@tyks.fi	Clinical Groups	**Cardiometabolic Diseases Group**
Jenni Aittokallio	Hospital District of Southwest Finland, Turku, Finland	jemato@utu.fi	Clinical Groups	**Cardiometabolic Diseases Group**
Mika Kähönen	Pirkanmaa Hospital District, Tampere, Finland	mika.kahonen@uta.fi	Clinical Groups	**Cardiometabolic Diseases Group**
Jussi Hernesniemi	Pirkanmaa Hospital District, Tampere, Finland	jussi.hernesniemi@tuni.fi	Clinical Groups	**Cardiometabolic Diseases Group**
Daniel Gordin	Hospital District of Helsinki and Uusimaa, Helsinki, Finland	daniel.gordin@hus.fi	Clinical Groups	**Cardiometabolic Diseases Group**
Juha Sinisalo	Hospital District of Helsinki and Uusimaa, Helsinki, Finland	juha.sinisalo@hus.fi	Clinical Groups	**Cardiometabolic Diseases Group**
Marja-Riitta Taskinen	Hospital District of Helsinki and Uusimaa, Helsinki, Finland	marja-riitta.taskinen@helsinki.fi	Clinical Groups	**Cardiometabolic Diseases Group**
Tiinamaija Tuomi	Hospital District of Helsinki and Uusimaa, Helsinki, Finland	tiinamaija.tuomi@hus.fi	Clinical Groups	**Cardiometabolic Diseases Group**
Timo Hiltunen	Hospital District of Helsinki and Uusimaa, Helsinki, Finland	timo.hiltunen@hus.fi	Clinical Groups	**Cardiometabolic Diseases Group**
Jari Laukkanen	Central Finland Health Care District, Jyväskylä, Finland	jari.laukkanen@ksshp.fi	Clinical Groups	**Cardiometabolic Diseases Group**
Amanda Elliott	Institute for Molecular Medicine Finland (FIMM), HiLIFE, University of Helsinki, Helsinki, Finland; Broad Institute, Cambridge, MA, USA and Massachusetts General Hospital, Boston, MA, USA	aelliott@broadinstitute.org	Clinical Groups	**Cardiometabolic Diseases Group**
Mary Pat Reeve	Institute for Molecular Medicine Finland (FIMM), HiLIFE, University of Helsinki, Helsink	mary.reeve@helsinki.fi	Clinical Groups	**Cardiometabolic Diseases Group**
Sanni Ruotsalainen	Institute for Molecular Medicine Finland (FIMM), HiLIFE, University of Helsinki, Helsink	sanni.ruotsalainen@helsinki.fi	Clinical Groups	**Cardiometabolic Diseases Group**
Dirk Paul	Astra Zeneca, Cambridge, United Kingdom	dirk.paul@astrazeneca.com	Clinical Groups	**Cardiometabolic Diseases Group**
Natalie Bowers	Genentech, San Francisco, CA, United States	bowersn1@gene.com	Clinical Groups	**Cardiometabolic Diseases Group**
Rion Pendergrass	Genentech, San Francisco, CA, United States	penders2@gene.com	Clinical Groups	**Cardiometabolic Diseases Group**
Audrey Chu	GlaxoSmithKline, Brentford, United Kingdom	audrey.y.chu@gsk.com	Clinical Groups	**Cardiometabolic Diseases Group**
Kirsi Auro	GlaxoSmithKline, Espoo, Finland	kirsi.m.auro@gsk.com	Clinical Groups	**Cardiometabolic Diseases Group**
Dermot Reilly	Janssen Research & Development, LLC, Boston, MA, United States	dreill11@its.jnj.com	Clinical Groups	**Cardiometabolic Diseases Group**
Mike Mendelson	Novartis, Boston, MA, United States	mike.mendelson@novartis.com	Clinical Groups	**Cardiometabolic Diseases Group**
Jaakko Parkkinen	Pfizer, New York, NY, United States	jaakko.parkkinen@pfizer.com	Clinical Groups	**Cardiometabolic Diseases Group**
Melissa Miller	Pfizer, New York, NY, United States	melissa.r.miller@pfizer.com	Clinical Groups	**Cardiometabolic Diseases Group**
Tuomo Meretoja	Department of Breast Surgery, Helsinki University Hospital Comprehensive Cancer Center and University of Helsinki, Helsinki, Finland	tuomo.meretoja@hus.fi	Clinical Groups	**Oncology Group**
Heikki Joensuu	Department of Oncology, Helsinki University Hospital Comprehensive Cancer Center and University of Helsinki, Helsinki, Finland	heikki.joensuu@hus.fi	Clinical Groups	**Oncology Group**
Olli Carpén	Hospital District of Helsinki and Uusimaa, Helsinki, Finland	olli.carpen@helsinki.fi	Clinical Groups	**Oncology Group**
Johanna Mattson	Hospital District of Helsinki and Uusimaa, Helsinki, Finland	johanna.mattson@hus.fi	Clinical Groups	**Oncology Group**
Eveliina Salminen	Hospital District of Helsinki and Uusimaa, Helsinki, Finland	eveliina.e.salminen@hus.fi	Clinical Groups	**Oncology Group**
Annika Auranen	Pirkanmaa Hospital District , Tampere, Finland	anaura@utu.fi	Clinical Groups	**Oncology Group**
Peeter Karihtala	Department of Oncology, Helsinki University Hospital Comprehensive Cancer Center and University of Helsinki, Helsinki, Finland	peeter.karihtala@hus.fi	Clinical Groups	**Oncology Group**
Päivi Auvinen	Northern Savo Hospital District, Kuopio, Finland	paivi.auvinen@kuh.fi	Clinical Groups	**Oncology Group**
Klaus Elenius	Hospital District of Southwest Finland, Turku, Finland	klaus.elenius@utu.fi	Clinical Groups	**Oncology Group**
Johanna Schleutker	Hospital District of Southwest Finland, Turku, Finland	johanna.schleutker@utu.fi	Clinical Groups	**Oncology Group**
Esa Pitkänen	Institute for Molecular Medicine Finland (FIMM), HiLIFE, University of Helsinki, Helsink	esa.pitkanen@helsinki.fi	Clinical Groups	**Oncology Group**
Nina Mars	Institute for Molecular Medicine Finland (FIMM), HiLIFE, University of Helsinki, Helsink	nina.mars@helsinki.fi	Clinical Groups	**Oncology Group**
Mark Daly	Institute for Molecular Medicine Finland (FIMM), HiLIFE, University of Helsinki, Helsinki, Finland; Broad Institute of MIT and Harvard; Massachusetts General Hospital	mark.daly@helsinki.fi	Clinical Groups	**Oncology Group**
Relja Popovic	Abbvie, Chicago, IL, United States	relja.popovic@abbvie.com	Clinical Groups	**Oncology Group**
Jeffrey Waring	Abbvie, Chicago, IL, United States	jeff.waring@abbvie.com	Clinical Groups	**Oncology Group**
Bridget Riley-Gillis	Abbvie, Chicago, IL, United States	bridget.rileygillis@abbvie.com	Clinical Groups	**Oncology Group**
Anne Lehtonen	Abbvie, Chicago, IL, United States	anne.lehtonen@abbvie.com	Clinical Groups	**Oncology Group**
Margarete Fabre	AstraZeneca, Cambridge, United Kingdom	margarete.fabre@astrazeneca.com	Clinical Groups	**Oncology Group**
Jennifer Schutzman	Genentech, San Francisco, CA, United States	schutzman.jennifer@gene.com	Clinical Groups	**Oncology Group**
Natalie Bowers	Genentech, San Francisco, CA, United States	bowersn1@gene.com	Clinical Groups	**Oncology Group**
Rion Pendergrass	Genentech, San Francisco, CA, United States	penders2@gene.com	Clinical Groups	**Oncology Group**
Diptee Kulkarni	GlaxoSmithKline, Brentford, United Kingdom	diptee.a.kulkarni@gsk.com	Clinical Groups	**Oncology Group**
Kirsi Auro	GlaxoSmithKline, Espoo, Finland	kirsi.m.auro@gsk.com	Clinical Groups	**Oncology Group**

Alessandro Porello	Janssen Research & Development, LLC, Spring House, PA, United States	APorrell@ITS.JNJ.com	Clinical Groups	Oncology Group
Andrey Loboda	Merck, Kenilworth, NJ, United States	andrey_loboda@merck.com	Clinical Groups	Oncology Group
Heli Lehtonen	Pfizer, New York, NY, United States	heli.lehtonen@pfizer.com	Clinical Groups	Oncology Group
Stefan McDonough	Pfizer, New York, NY, United States	stefan.McDonough@pfizer.com	Clinical Groups	Oncology Group
Sauli Vuoti	Janssen-Cilag Oy, Espoo, Finland	svuoti@its.jnj.com	Clinical Groups	Oncology Group
Kai Kaarniranta	Northern Savo Hospital District, Kuopio, Finland; Department of Molecular Genetics, University of Lodz, Lodz, Poland	kai.kaarniranta@uef.fi	Clinical Groups	Opthalmology Group
Joni A Turunen	Helsinki University Hospital and University of Helsinki, Helsinki, Finland; Eye Genetics Group, Folkhälsan Research Center, Helsinki, Finland	joni.turunen@helsinki.fi	Clinical Groups	Opthalmology Group
Terhi Ollila	Hospital District of Helsinki and Uusimaa, Helsinki, Finland	terhi.ollila@hus.fi	Clinical Groups	Opthalmology Group
Hannu Uusitalo	Pirkanmaa Hospital District, Tampere, Finland	hannu.uusitalo@tuni.fi	Clinical Groups	Opthalmology Group
Juha Karjalainen	Institute for Molecular Medicine Finland (FIMM), HiLIFE, University of Helsinki, Helsink	juha.karjalainen@helsinki.fi	Clinical Groups	Opthalmology Group
Esa Pitkänen	Institute for Molecular Medicine Finland (FIMM), HiLIFE, University of Helsinki, Helsink	esa.pitkanen@helsinki.fi	Clinical Groups	Opthalmology Group
Mengzhen Liu	Abbvie, Chicago, IL, United States	mengzhen.liu@abbvie.com	Clinical Groups	Opthalmology Group
Heiko Runz	Biogen, Cambridge, MA, United States	heiko.runz@biogen.com	Clinical Groups	Opthalmology Group
Stephanie Loomis	Biogen, Cambridge, MA, United States	stephanie.loomis@biogen.com	Clinical Groups	Opthalmology Group
Erich Strauss	Genentech, San Francisco, CA, United States	strauss.erich@gene.com	Clinical Groups	Opthalmology Group
Natalie Bowers	Genentech, San Francisco, CA, United States	bowersn1@gene.com	Clinical Groups	Opthalmology Group
Hao Chen	Genentech, San Francisco, CA, United States	haoc@gene.com	Clinical Groups	Opthalmology Group
Rion Pendergrass	Genentech, San Francisco, CA, United States	penders2@gene.com	Clinical Groups	Opthalmology Group
Kaisa Tasanen	Northern Ostrobothnia Hospital District, Oulu, Finland	kaisa.tasanen-maatta@oulu.fi	Clinical Groups	Dermatology Group
Laura Huilaja	Northern Ostrobothnia Hospital District, Oulu, Finland	laura.huilaja@oulu.fi	Clinical Groups	Dermatology Group
Katariina Hannula-Joupp	Hospital District of Helsinki and Uusimaa, Helsinki, Finland	katariina.hannula-jouppi@hus.fi	Clinical Groups	Dermatology Group
Teea Salmi	Pirkanmaa Hospital District, Tampere, Finland	teea.salmi@pshp.fi	Clinical Groups	Dermatology Group
Sirkku Peltonen	Hospital District of Southwest Finland, Turku, Finland	sipelto@utu.fi	Clinical Groups	Dermatology Group
Leena Koulu	Hospital District of Southwest Finland, Turku, Finland	leena.koulu@tyks.fi	Clinical Groups	Dermatology Group
Nizar Smaoui	Abbvie, Chicago, IL, United States	nizar.smaoui@abbvie.com	Clinical Groups	Dermatology Group
Fedik Rahimov	Abbvie, Chicago, IL, United States	fedik.rahimov@abbvie.com	Clinical Groups	Dermatology Group
Anne Lehtonen	Abbvie, Chicago, IL, United States	anne.lehtonen@abbvie.com	Clinical Groups	Dermatology Group
David Choy	Genentech, San Francisco, CA, United States	choy.david@gene.com	Clinical Groups	Dermatology Group
Rion Pendergrass	Genentech, San Francisco, CA, United States	penders2@gene.com	Clinical Groups	Dermatology Group
Dawn Waterworth	Janssen Research & Development, LLC, Spring House, PA, United States	dwaterwo@its.jnj.com	Clinical Groups	Dermatology Group
Kirsi Kalpala	Pfizer, New York, NY, United States	kirsi.kalpala@pfizer.com	Clinical Groups	Dermatology Group
Ying Wu	Pfizer, New York, NY, United States	ying.wu3@pfizer.com	Clinical Groups	Dermatology Group
Pirkko Pussinen	Hospital District of Helsinki and Uusimaa, Helsinki, Finland	pirkko.pussinen@helsinki.fi	Clinical Groups	Odontology Group
Aino Salminen	Hospital District of Helsinki and Uusimaa, Helsinki, Finland	aino.m.salminen@helsinki.fi	Clinical Groups	Odontology Group
Tuula Salo	Hospital District of Helsinki and Uusimaa, Helsinki, Finland	tuula.salo@helsinki.fi	Clinical Groups	Odontology Group
David Rice	Hospital District of Helsinki and Uusimaa, Helsinki, Finland	david.rice@helsinki.fi	Clinical Groups	Odontology Group
Pekka Nieminen	Hospital District of Helsinki and Uusimaa, Helsinki, Finland	pekka.nieminen@helsinki.fi	Clinical Groups	Odontology Group
Ulla Palotie	Hospital District of Helsinki and Uusimaa, Helsinki, Finland	ulla.palotie@helsinki.fi	Clinical Groups	Odontology Group
Maria Siponen	Northern Savo Hospital District, Kuopio, Finland	maria.siponen@uef.fi	Clinical Groups	Odontology Group
Liisa Suominen	Northern Savo Hospital District, Kuopio, Finland	liisa.suominen@uef.fi	Clinical Groups	Odontology Group
Päivi Mäntylä	Northern Savo Hospital District, Kuopio, Finland	paivi.mantyla@uef.fi	Clinical Groups	Odontology Group
Ulvi Gursoy	Hospital District of Southwest Finland, Turku, Finland	ulvi.gursoy@utu.fi	Clinical Groups	Odontology Group
Vuokko Anttonen	Northern Ostrobothnia Hospital District, Oulu, Finland	vuokko.anttonen@oulu.fi	Clinical Groups	Odontology Group
Kirsi Sipilä	Research Unit of Oral Health Sciences Faculty of Medicine, University of Oulu, Oulu, Finland; Medical Research Center, Oulu, Oulu University Hospital and University of Oulu, Oulu, Finland	kirsi.sipila@oulu.fi	Clinical Groups	Odontology Group
Rion Pendergrass	Genentech, San Francisco, CA, United States	pendergrass.sarah@gene.com	Clinical Groups	Odontology Group
Hannele Laivuori	Institute for Molecular Medicine Finland (FIMM), HiLIFE, University of Helsinki, Helsink	hannele.laivuori@helsinki.fi	Clinical Groups	Women's Health and Reproduction Group
Venla Kurra	Pirkanmaa Hospital District, Tampere, Finland	venla.kurra@tuni.fi	Clinical Groups	Women's Health and Reproduction Group
Laura Kotaniemi-Talone	Pirkanmaa Hospital District, Tampere, Finland	laura.kotaniemi-talonen@tuni.fi	Clinical Groups	Women's Health and Reproduction Group
Oskari Heikinheimo	Hospital District of Helsinki and Uusimaa, Helsinki, Finland	oskari.heikinheimo@helsinki.fi	Clinical Groups	Women's Health and Reproduction Group
Ilkka Kalliala	Hospital District of Helsinki and Uusimaa, Helsinki, Finland	ilkka.kalliala@hus.fi	Clinical Groups	Women's Health and Reproduction Group
Lauri Aaltonen	Hospital District of Helsinki and Uusimaa, Helsinki, Finland	lauri.aaltonen@helsinki.fi	Clinical Groups	Women's Health and Reproduction Group
Varpu Jokimaa	Hospital District of Southwest Finland, Turku, Finland	varpu.jokimaa@utu.fi	Clinical Groups	Women's Health and Reproduction Group
Johannes Kettunen	Northern Ostrobothnia Hospital District, Oulu, Finland	Johannes.Kettunen@oulu.fi	Clinical Groups	Women's Health and Reproduction Group
Marja Vääräsmäki	Northern Ostrobothnia Hospital District, Oulu, Finland	marja.vaarasmaki@oulu.fi	Clinical Groups	Women's Health and Reproduction Group
Outi Uimari	Northern Ostrobothnia Hospital District, Oulu, Finland	outi.uimari@oulu.fi	Clinical Groups	Women's Health and Reproduction Group
Laure Morin-Papunen	Northern Ostrobothnia Hospital District, Oulu, Finland	lmp@cc.oulu.fi	Clinical Groups	Women's Health and Reproduction Group
Maarit Niinimäki	Northern Ostrobothnia Hospital District, Oulu, Finland	maarit.niinimaki@oulu.fi	Clinical Groups	Women's Health and Reproduction Group
Terhi Piltonen	Northern Ostrobothnia Hospital District, Oulu, Finland	terhi.piltonen@oulu.fi	Clinical Groups	Women's Health and Reproduction Group
Katja Kivinen	Institute for Molecular Medicine Finland (FIMM), HiLIFE, University of Helsinki, Helsink	katja.kivinen@helsinki.fi	Clinical Groups	Women's Health and Reproduction Group
Elisabeth Widen	Institute for Molecular Medicine Finland (FIMM), HiLIFE, University of Helsinki, Helsink	elisabeth.widen@helsinki.fi	Clinical Groups	Women's Health and Reproduction Group
Taru Tukiainen	Institute for Molecular Medicine Finland (FIMM), HiLIFE, University of Helsinki, Helsink	taru.tukiainen@helsinki.fi	Clinical Groups	Women's Health and Reproduction Group
Mary Pat Reeve	Institute for Molecular Medicine Finland (FIMM), HiLIFE, University of Helsinki, Helsink	mary.reeve@helsinki.fi	Clinical Groups	Women's Health and Reproduction Group
Mark Daly	Institute for Molecular Medicine Finland (FIMM), HiLIFE, University of Helsinki, Helsinki, Finland; Broad Institute of MIT and Harvard; Massachusetts General Hospital	mark.daly@helsinki.fi	Clinical Groups	Women's Health and Reproduction Group
Niko Välimäki	University of Helsinki, Helsinki, Finland	niko.valimaki@helsinki.fi	Clinical Groups	Women's Health and Reproduction Group
Eija Laakkonen	University of Jyväskylä, Jyväskylä, Finland	eija.k.laakkonen@jyu.fi	Clinical Groups	Women's Health and Reproduction Group
Jaakko Tyrmi	University of Oulu, Oulu, Finland / University of Tampere, Tampere, Finland	jaakko.tyrmi@oulu.fi	Clinical Groups	Women's Health and Reproduction Group
Heidi Silven	University of Oulu, Oulu, Finland	heidi.silven@student.oulu.fi	Clinical Groups	Women's Health and Reproduction Group
Eeva Sliz	University of Oulu, Oulu, Finland	eeva.sliz@oulu.fi	Clinical Groups	Women's Health and Reproduction Group
Riikka Arffman	University of Oulu, Oulu, Finland	riikka.arffman@oulu.fi	Clinical Groups	Women's Health and Reproduction Group
Susanna Savukoski	University of Oulu, Oulu, Finland	susanna.savukoski@oulu.fi	Clinical Groups	Women's Health and Reproduction Group
Triin Laisk	Estonian biobank, Tartu, Estonia	triin.laisk@ut.ee	Clinical Groups	Women's Health and Reproduction Group
Natalia Pujol	Estonian biobank, Tartu, Estonia	natalia.pujolgualdo@oulu.fi	Clinical Groups	Women's Health and Reproduction Group
Mengzhen Liu	Abbvie, Chicago, IL, United States	mengzhen.liu@abbvie.com	Clinical Groups	Women's Health and Reproduction Group
Bridget Riley-Gillis	Abbvie, Chicago, IL, United States	bridget.rileygillis@abbvie.com	Clinical Groups	Women's Health and Reproduction Group
Rion Pendergrass	Genentech, San Francisco, CA, United States	penders2@gene.com	Clinical Groups	Women's Health and Reproduction Group
Janet Kumar	GlaxoSmithKline, Collegeville, PA, United States	janet.x.kumar@gsk.com	Clinical Groups	Women's Health and Reproduction Group
Kirsi Auro	GlaxoSmithKline, Espoo, Finland	kirsi.m.auro@gsk.com	Clinical Groups	Women's Health and Reproduction Group
Iiris Hovatta	University of Helsinki, Finland	iiris.hovatta@helsinki.fi	Clinical Groups	Depression group
Chia-Yen Chen	Biogen, Cambridge, MA, United States	chiayen.chen@biogen.com	Clinical Groups	Depression group
Erkki Isometsä	Hospital District of Helsinki and Uusimaa, Helsinki, Finland	erkki.isometsa@hus.fi	Clinical Groups	Depression group
Hanna Ollila	Institute for Molecular Medicine Finland (FIMM), HiLIFE, University of Helsinki, Helsink	hanna.m.ollila@helsinki.fi	Clinical Groups	Depression group
Jaana Suvisaari	Finnish Institute for Health and Welfare (THL), Helsinki, Finland	jaana.suvisaari@thl.fi	Clinical Groups	Depression group
Antti Mäkitie	Department of Otorhinolaryngology - Head and Neck Surgery, University of Helsinki and Helsinki University Hospital, Helsinki, Finland	antti.makitie@helsinki.fi	Clinical Groups	ENT (ear, nose and throath) Group
Argyro Bizaki-Vallaskang	Pirkanmaa Hospital District, Tampere, Finland	argyro.bizaki-vallaskangas@tuni.fi	Clinical Groups	ENT (ear, nose and throath) Group
Sanna Toppila-Salmi	University of Eastern Finland and Kuopio University Hospital, Department of Otorhinolaryngology, Kuopio, Finland and Department of Allergy, Helsinki University Hospital and University of Helsinki, Helsinki, Finland	sanna.salmi@helsinki.fi	Clinical Groups	ENT (ear, nose and throath) Group
Tytti Willberg	Hospital District of Southwest Finland, Turku, Finland	tytti.willberg@tyks.fi	Clinical Groups	ENT (ear, nose and throath) Group
Elmo Saarentaus	Institute for Molecular Medicine Finland (FIMM), HiLIFE, University of Helsinki, Helsink	elmo.saarentaus@helsinki.fi	Clinical Groups	ENT (ear, nose and throath) Group
Antti Aarnisalo	Hospital District of Helsinki and Uusimaa, Helsinki, Finland	antti.aarnisalo@hus.fi	Clinical Groups	ENT (ear, nose and throath) Group
Eveliina Salminen	Hospital District of Helsinki and Uusimaa, Helsinki, Finland	eveliina.e.salminen@hus.fi	Clinical Groups	ENT (ear, nose and throath) Group
Elisa Rahikkala	Northern Ostrobothnia Hospital District, Oulu, Finland	elisa.rahikkala@ppshp.fi	Clinical Groups	ENT (ear, nose and throath) Group
Johannes Kettunen	Northern Ostrobothnia Hospital District, Oulu, Finland	johannes.kettunen@oulu.fi	Clinical Groups	ENT (ear, nose and throath) Group
Kristiina Aittomäki	Department of Medical Genetics, Helsinki University Central Hospital, Helsinki, Finland	kristiina.aittomaki@helsinki.fi	Clinical Groups	POI (premature ovarian failure) Group
Fredrik Åberg	Transplantation and Liver Surgery Clinic, Helsinki University Hospital, Helsinki University, Helsinki, Finland	fredrik.aberg@helsinki.fi	Clinical Groups	LiverScore Group
Mitja Kurki	Institute for Molecular Medicine Finland (FIMM), HiLIFE, University of Helsinki, Helsinki, Finland; Broad Institute, Cambridge, MA, United States	mkurki@broadinstitute.org	FinnGen Analysis working g	FinnGen Analysis working group
Samuli Ripatti	Institute for Molecular Medicine Finland (FIMM), HiLIFE, University of Helsinki, Helsink	samuli.ripatti@helsinki.fi	FinnGen Analysis working g	FinnGen Analysis working group
Mark Daly	Institute for Molecular Medicine, Finland (FIMM), HiLIFE, University of Helsinki, Helsinki, Finland; Broad Institute of MIT and Harvard; Massachusetts General Hospital	mark.daly@helsinki.fi	FinnGen Analysis working g	FinnGen Analysis working group
Juha Karjalainen	Institute for Molecular Medicine Finland (FIMM), HiLIFE, University of Helsinki, Helsink	juha.karjalainen@helsinki.fi	FinnGen Analysis working g	FinnGen Analysis working group
Aki Havulinna	Institute for Molecular Medicine Finland (FIMM), HiLIFE, University of Helsinki, Helsin	aki.havulinna@helsinki.fi	FinnGen Analysis working g	FinnGen Analysis working group
Juha Mehtonen	Institute for Molecular Medicine Finland (FIMM), HiLIFE, University of Helsinki, Helsink	juha.mehtonen@helsinki.fi	FinnGen Analysis working g	FinnGen Analysis working group
Priit Palta	Institute for Molecular Medicine Finland (FIMM), HiLIFE, University of Helsinki, Helsink	priit.palta@helsinki.fi	FinnGen Analysis working g	FinnGen Analysis working group
Shabbeer Hassan	Institute for Molecular Medicine Finland (FIMM), HiLIFE, University of Helsinki, Helsink	shabbeer.hassan@helsinki.fi	FinnGen Analysis working g	FinnGen Analysis working group
Pietro Della Briotta Paro	Institute for Molecular Medicine Finland (FIMM), HiLIFE, University of Helsinki, Helsink	pietro.dellabriottaparolo@helsinki.fi	FinnGen Analysis working g	FinnGen Analysis working group
Wei Zhou	Broad Institute, Cambridge, MA, United States	wzhou@broadinstitute.org	FinnGen Analysis working g	FinnGen Analysis working group
Mutaamba Maasha	Broad Institute, Cambridge, MA, United States	mmaasha@broadinstitute.org	FinnGen Analysis working g	FinnGen Analysis working group
Shabbeer Hassan	Institute for Molecular Medicine Finland (FIMM), HiLIFE, University of Helsinki, Helsink	shabbeer.hassan@helsinki.fi	FinnGen Analysis working g	FinnGen Analysis working group
Susanna Lemmelä	Institute for Molecular Medicine Finland (FIMM), HiLIFE, University of Helsinki, Helsink	susanna.lemmela@helsinki.fi	FinnGen Analysis working g	FinnGen Analysis working group
Manuel Rivas	University of Stanford, Stanford, CA, United States	mrivas@stanford.edu	FinnGen Analysis working g	FinnGen Analysis working group
Aarno Palotie	Institute for Molecular Medicine Finland (FIMM), HiLIFE, University of Helsinki, Helsink	aarno.palotie@helsinki.fi	FinnGen Analysis working g	FinnGen Analysis working group
Aoxing Liu	Institute for Molecular Medicine Finland (FIMM), HiLIFE, University of Helsinki, Helsink	aoxing.liu@helsinki.fi	FinnGen Analysis working g	FinnGen Analysis working group
Arto Lehisto	Institute for Molecular Medicine Finland (FIMM), HiLIFE, University of Helsinki, Helsink	arto.lehisto@helsinki.fi	FinnGen Analysis working g	FinnGen Analysis working group
Andrea Ganna	Institute for Molecular Medicine Finland (FIMM), HiLIFE, University of Helsinki, Helsink	aganna@broadinstitute.org	FinnGen Analysis working g	FinnGen Analysis working group
Vincent Llorens	Institute for Molecular Medicine Finland (FIMM), HiLIFE, University of Helsinki, Helsink	vincent.llorens@helsinki.fi	FinnGen Analysis working g	FinnGen Analysis working group
Hannele Laivuori	Institute for Molecular Medicine Finland (FIMM), HiLIFE, University of Helsinki, Helsink	hannele.laivuori@helsinki.fi	FinnGen Analysis working g	FinnGen Analysis working group

Taru Tukiainen	Institute for Molecular Medicine Finland (FIMM), HiLIFE, University of Helsinki, Helsink	taru.tukiainen@helsinki.fi	FinnGen Analysis working g	FinnGen Analysis working group
Mary Pat Reeve	Institute for Molecular Medicine Finland (FIMM), HiLIFE, University of Helsinki, Helsink	mary.reeve@helsinki.fi	FinnGen Analysis working g	FinnGen Analysis working group
Henrike Heyne	Institute for Molecular Medicine Finland (FIMM), HiLIFE, University of Helsinki, Helsink	hheyne@broadinstitute.org	FinnGen Analysis working g	FinnGen Analysis working group
Nina Mars	Institute for Molecular Medicine Finland (FIMM), HiLIFE, University of Helsinki, Helsink	nina.mars@helsinki.fi	FinnGen Analysis working g	FinnGen Analysis working group
Joel Rämö	Institute for Molecular Medicine Finland (FIMM), HiLIFE, University of Helsinki, Helsink	joel.ramo@helsinki.fi	FinnGen Analysis working g	FinnGen Analysis working group
Elmo Saarentaus	Institute for Molecular Medicine Finland (FIMM), HiLIFE, University of Helsinki, Helsink	elmo.saarentaus@helsinki.fi	FinnGen Analysis working g	FinnGen Analysis working group
Hanna Ollila	Institute for Molecular Medicine Finland (FIMM), HiLIFE, University of Helsinki, Helsink	hanna.m.ollila@helsinki.fi	FinnGen Analysis working g	FinnGen Analysis working group
Rodos Rodosthenous	Institute for Molecular Medicine Finland (FIMM), HiLIFE, University of Helsinki, Helsink	rodos.rodosthenous@helsinki.fi	FinnGen Analysis working g	FinnGen Analysis working group
Satu Strausz	Institute for Molecular Medicine Finland (FIMM), HiLIFE, University of Helsinki, Helsink	satu.strausz@helsinki.fi	FinnGen Analysis working g	FinnGen Analysis working group
Tuula Palotie	University of Helsinki and Hospital District of Helsinki and Uusimaa, Helsinki, Finland	tuula.palotie@helsinki.fi	FinnGen Analysis working g	FinnGen Analysis working group
Kimmo Palin	University of Helsinki, Helsinki, Finland	kimmo.palin@helsinki.fi	FinnGen Analysis working g	FinnGen Analysis working group
Javier Garcia-Tabuenca	University of Tampere, Tampere, Finland	javier.graciatabuenca@tuni.fi	FinnGen Analysis working g	FinnGen Analysis working group
Harri Siirtola	University of Tampere, Tampere, Finland	harri.siirtola@tuni.fi	FinnGen Analysis working g	FinnGen Analysis working group
Tuomo Kiiskinen	Institute for Molecular Medicine Finland (FIMM), HiLIFE, University of Helsinki, Helsink	tuomo.kiiskinen@helsinki.fi	FinnGen Analysis working g	FinnGen Analysis working group
Jiwoo Lee	Institute for Molecular Medicine Finland (FIMM), HiLIFE, University of Helsinki, Helsinki, Finland; Broad Institute, Cambridge, MA, United States	jiwoo.lee@helsinki.fi	FinnGen Analysis working g	FinnGen Analysis working group
Kristin Tsuo	Institute for Molecular Medicine Finland (FIMM), HiLIFE, University of Helsinki, Helsinki, Finland; Broad Institute, Cambridge, MA, United States	kristintsuo@fas.harvard.edu	FinnGen Analysis working g	FinnGen Analysis working group
Amanda Elliott	Institute for Molecular Medicine Finland (FIMM), HiLIFE, University of Helsinki, Helsinki, Finland; Broad Institute, Cambridge, MA, USA and Massachusetts General Hospital, Boston, MA, USA	aelliott@broadinstitute.org	FinnGen Analysis working g	FinnGen Analysis working group
Kati Kristiansson	THL Biobank / Finnish Institute for Health and Welfare (THL), Helsinki, Finland	kati.kristiansson@thl.fi	FinnGen Analysis working g	FinnGen Analysis working group
Mikko Arvas	Finnish Red Cross Blood Service / Finnish Hematology Registry and Clinical Biobank, Helsinki, Finland	mikko.arvas@veripalvelu.fi	FinnGen Analysis working g	FinnGen Analysis working group
Kati Hyvärinen	Finnish Red Cross Blood Service, Helsinki, Finland	kati.hyvarinen@veripalvelu.fi	FinnGen Analysis working g	FinnGen Analysis working group
Jarmo Ritari	Finnish Red Cross Blood Service, Helsinki, Finland	jarmo.ritari@veripalvelu.fi	FinnGen Analysis working g	FinnGen Analysis working group
Olli Carpén	Helsinki Biobank / Helsinki University and Hospital District of Helsinki and Uusimaa, Helsinki	olli.carpen@helsinki.fi	FinnGen Analysis working g	FinnGen Analysis working group
Johannes Kettunen	Northern Finland Biobank Borealis / University of Oulu / Northern Ostrobothnia Hospital District, Oulu, Finland	johannes.kettunen@oulu.fi	FinnGen Analysis working g	FinnGen Analysis working group
Katri Pylkäs	University of Oulu, Oulu, Finland	katri.pylkas@oulu.fi	FinnGen Analysis working g	FinnGen Analysis working group
Eeva Sliz	University of Oulu, Oulu, Finland	eeva.sliz@oulu.fi	FinnGen Analysis working g	FinnGen Analysis working group
Minna Karjalainen	University of Oulu, Oulu, Finland	minna.k.karjalainen@oulu.fi	FinnGen Analysis working g	FinnGen Analysis working group
Tuomo Mantere	Northern Finland Biobank Borealis / University of Oulu / Northern Ostrobothnia Hospital District, Oulu, Finland	tuomo.mantere@oulu.fi	FinnGen Analysis working g	FinnGen Analysis working group
Eeva Kangasniemi	Finnish Clinical Biobank Tampere / University of Tampere / Pirkanmaa Hospital District, Tampere, Finland	eeva.kangasniemi@pshp.fi	FinnGen Analysis working g	FinnGen Analysis working group
Sami Heikkinen	University of Eastern Finland, Kuopio, Finland	sami.heikkinen@uef.fi	FinnGen Analysis working g	FinnGen Analysis working group
Arto Mannermaa	Biobank of Eastern Finland / University of Eastern Finland / Northern Savo Hospital District, Kuopio, Finland	arto.mannermaa@uef.fi	FinnGen Analysis working g	FinnGen Analysis working group
Eija Laakkonen	University of Jyväskylä, Jyväskylä, Finland	eija.k.laakkonen@jyu.fi	FinnGen Analysis working g	FinnGen Analysis working group
Nina Pitkänen	Auria Biobank / University of Turku / Hospital District of Southwest Finland, Turku, Finland	Niina.Pitkanen@tyks.fi	FinnGen Analysis working g	FinnGen Analysis working group
Samuel Lessard	Translational Sciences, Sanofi R&D, Framingham, MA, USA	samuel.lessard@sanofi.com	FinnGen Analysis working g	FinnGen Analysis working group
Clément Chatelain	Translational Sciences, Sanofi R&D, Framingham, MA, USA	clement.chatelain@sanofi.com	FinnGen Analysis working g	FinnGen Analysis working group
Lila Kallio	Auria Biobank / University of Turku / Hospital District of Southwest Finland, Turku, Finland	Lila.Kallio@tyks.fi	Biobank directors	Biobank directors
Tiina Wahlfors	THL Biobank / Finnish Institute for Health and Welfare (THL), Helsinki, Finland	tiina.wahlfors@thl.fi	Biobank directors	Biobank directors
Jukka Partanen	Finnish Red Cross Blood Service / Finnish Hematology Registry and Clinical Biobank, Helsinki, Finland	jukka.partanen@veripalvelu.fi	Biobank directors	Biobank directors
Eero Punkka	Helsinki Biobank / Helsinki University and Hospital District of Helsinki and Uusimaa, Helsinki	eero.punkka@hus.fi	Biobank directors	Biobank directors
Raisa Serpi	Northern Finland Biobank Borealis / University of Oulu / Northern Ostrobothnia Hospital District, Oulu, Finland	raisa.serpi@ppshp.fi	Biobank directors	Biobank directors
Sanna Siltanen	Finnish Clinical Biobank Tampere / University of Tampere / Pirkanmaa Hospital District, Tampere, Finland	sanna.siltanen@pshp.fi	Biobank directors	Biobank directors
Veli-Matti Kosma	Biobank of Eastern Finland / University of Eastern Finland / Northern Savo Hospital District, Kuopio, Finland	veli-matti.kosma@uef.fi	Biobank directors	Biobank directors
Teijo Kuopio	Central Finland Biobank / University of Jyväskylä / Central Finland Health Care District, Jyväskylä, Finland	teijo.kuopio@ksshp.fi	Biobank directors	Biobank directors
Anu Jalanko	Institute for Molecular Medicine Finland (FIMM), HiLIFE, University of Helsinki, Helsink	anu.jalanko@helsinki.fi	FinnGen Teams	**Administration**
Huei-Yi Shen	Institute for Molecular Medicine Finland (FIMM), HiLIFE, University of Helsinki, Helsink	huei-yi.shen@helsinki.fi	FinnGen Teams	**Administration**
Risto Kajanne	Institute for Molecular Medicine Finland (FIMM), HiLIFE, University of Helsinki, Helsink	risto.kajanne@helsinki.fi	FinnGen Teams	**Administration**
Mervi Aavikko	Institute for Molecular Medicine Finland (FIMM), HiLIFE, University of Helsinki, Helsink	mervi.aavikko@helsinki.fi	FinnGen Teams	**Administration**
Helen Cooper	Institute for Molecular Medicine Finland (FIMM), HiLIFE, University of Helsinki, Helsink	helen.cooper@helsinki.fi	FinnGen Teams	**Administration**
Denise Öller	Institute for Molecular Medicine Finland (FIMM), HiLIFE, University of Helsinki, Helsink	denise.oller@helsinki.fi	FinnGen Teams	**Administration**
Rasko Leinonen	Institute for Molecular Medicine Finland (FIMM), HiLIFE, University of Helsinki, Helsink	rasko@ebi.ac.uk	FinnGen Teams	**Administration**
Henna Palin	Finnish Clinical Biobank Tampere / University of Tampere / Pirkanmaa Hospital District, Tampere, Finland	henna.palin@pshp.fi	FinnGen Teams	**Administration**
Malla-Maria Linna	Helsinki Biobank / Helsinki University and Hospital District of Helsinki and Uusimaa, Helsinki	malla-maria.linna@hus.fi	FinnGen Teams	**Administration**
Mitja Kurki	Institute for Molecular Medicine Finland (FIMM), HiLIFE, University of Helsinki, Helsinki, Finland; Broad Institute, Cambridge, MA, United States	mkurki@broadinstitute.org	FinnGen Teams	**Analysis**
Juha Karjalainen	Institute for Molecular Medicine Finland (FIMM), HiLIFE, University of Helsinki, Helsink	juha.karjalainen@helsinki.fi	FinnGen Teams	**Analysis**
Pietro Della Briotta Paro	Institute for Molecular Medicine Finland (FIMM), HiLIFE, University of Helsinki, Helsink	pietro.dellabriottaparolo@helsinki.fi	FinnGen Teams	**Analysis**
Arto Lehisto	Institute for Molecular Medicine Finland (FIMM), HiLIFE, University of Helsinki, Helsink	arto.lehisto@helsinki.fi	FinnGen Teams	**Analysis**
Juha Mehtonen	Institute for Molecular Medicine Finland (FIMM), HiLIFE, University of Helsinki, Helsink	juha.mehtonen@helsinki.fi	FinnGen Teams	**Analysis**
Wei Zhou	Broad Institute, Cambridge, MA, United States	wzhou@broadinstitute.org	FinnGen Teams	**Analysis**
Masahiro Kanai	Broad Institute, Cambridge, MA, United States	mkanai@broadinstitute.org	FinnGen Teams	**Analysis**
Mutaamba Maasha	Broad Institute, Cambridge, MA, United States	mmaasha@broadinstitute.org	FinnGen Teams	**Analysis**
Zhili Zheng	Broad Institute, Cambridge, MA, United States	zhengzhi@broadinstitute.org	FinnGen Teams	**Analysis**
Hannele Laivuori	Institute for Molecular Medicine Finland (FIMM), HiLIFE, University of Helsinki, Helsink	hannele.laivuori@helsinki.fi	FinnGen Teams	**Clinical Endpoint Development**
Aki Havulinna	Institute for Molecular Medicine Finland (FIMM), HiLIFE, University of Helsinki, Helsin	aki.havulinna@helsinki.fi	FinnGen Teams	**Clinical Endpoint Development**
Susanna Lemmelä	Institute for Molecular Medicine Finland (FIMM), HiLIFE, University of Helsinki, Helsink	susanna.lemmela@helsinki.fi	FinnGen Teams	**Clinical Endpoint Development**
Tuomo Kiiskinen	Institute for Molecular Medicine Finland (FIMM), HiLIFE, University of Helsinki, Helsink	tuomo.kiiskinen@helsinki.fi	FinnGen Teams	**Clinical Endpoint Development**
L. Elisa Lahtela	Institute for Molecular Medicine Finland (FIMM), HiLIFE, University of Helsinki, Helsink	laura.lahtela@helsinki.fi	FinnGen Teams	**Clinical Endpoint Development**
Mari Kaunisto	Institute for Molecular Medicine Finland (FIMM), HiLIFE, University of Helsinki, Helsink	mari.kaunisto@helsinki.fi	FinnGen Teams	**Communication**
Elina Kilpeläinen	Institute for Molecular Medicine Finland (FIMM), HiLIFE, University of Helsinki, Helsink	elina.kilpelainen@helsinki.fi	FinnGen Teams	**E-Science**
Timo P. Sipilä	Institute for Molecular Medicine Finland (FIMM), HiLIFE, University of Helsinki, Helsink	timo.p.sipila@helsinki.fi	FinnGen Teams	**E-Science**
Oluwaseun Alexander D	Institute for Molecular Medicine Finland (FIMM), HiLIFE, University of Helsinki, Helsink	alexander.dada@helsinki.fi	FinnGen Teams	**E-Science**
Awaisa Ghazal	Institute for Molecular Medicine Finland (FIMM), HiLIFE, University of Helsinki, Helsink	awaisa.ghazal@helsinki.fi	FinnGen Teams	**E-Science**
Anastasia Kytölä	Institute for Molecular Medicine Finland (FIMM), HiLIFE, University of Helsinki, Helsink	anastasia.shcherban@helsinki.fi	FinnGen Teams	**E-Science**
Rigbe Weldatsadik	Institute for Molecular Medicine Finland (FIMM), HiLIFE, University of Helsinki, Helsink	rigbe.weldatsadik@helsinki.fi	FinnGen Teams	**E-Science**
Sanni Ruotsalainen	Institute for Molecular Medicine Finland (FIMM), HiLIFE, University of Helsinki, Helsink	sanni.ruotsalainen@helsinki.fi	FinnGen Teams	**E-Science**
Kati Donner	Institute for Molecular Medicine Finland (FIMM), HiLIFE, University of Helsinki, Helsink	kati.donner@helsinki.fi	FinnGen Teams	**Genotyping**
Timo P. Sipilä	Institute for Molecular Medicine Finland (FIMM), HiLIFE, University of Helsinki, Helsink	timo.p.sipila@helsinki.fi	FinnGen Teams	**Genotyping**
Anu Loukola	Helsinki Biobank / Helsinki University and Hospital District of Helsinki and Uusimaa, Helsinki	anu.loukola@hus.fi	FinnGen Teams	**Sample Collection Coordination**
Päivi Laiho	THL Biobank / Finnish Institute for Health and Welfare (THL), Helsinki, Finland	paivi.laiho@thl.fi	FinnGen Teams	**Sample Logistics**
Tuuli Sistonen	THL Biobank / Finnish Institute for Health and Welfare (THL), Helsinki, Finland	tuuli.sistonen@thl.fi	FinnGen Teams	**Sample Logistics**
Essi Kaiharju	THL Biobank / Finnish Institute for Health and Welfare (THL), Helsinki, Finland	essi.kaiharju@thl.fi	FinnGen Teams	**Sample Logistics**
Markku Laukkanen	THL Biobank / Finnish Institute for Health and Welfare (THL), Helsinki, Finland	markku.laukkanen@thl.fi	FinnGen Teams	**Sample Logistics**
Elina Järvensivu	THL Biobank / Finnish Institute for Health and Welfare (THL), Helsinki, Finland	elina.jarvensivu@thl.fi	FinnGen Teams	**Sample Logistics**
Sini Lähteenmäki	THL Biobank / Finnish Institute for Health and Welfare (THL), Helsinki, Finland	sini.lahteenmaki@thl.fi	FinnGen Teams	**Sample Logistics**
Lotta Männikkö	THL Biobank / Finnish Institute for Health and Welfare (THL), Helsinki, Finland	lotta.mannikko@thl.fi	FinnGen Teams	**Sample Logistics**
Regis Wong	THL Biobank / Finnish Institute for Health and Welfare (THL), Helsinki, Finland	regis.wong@thl.fi	FinnGen Teams	**Sample Logistics**
Auli Toivola	THL Biobank / Finnish Institute for Health and Welfare (THL), Helsinki, Finland	auli.toivola@thl.fi	FinnGen Teams	**Sample Logistics**
Minna Brunfeldt	THL Biobank / Finnish Institute for Health and Welfare (THL), Helsinki, Finland	minna.brunfeldt@thl.fi	FinnGen Teams	**Registry Data Operations**
Hannele Mattsson	THL Biobank / Finnish Institute for Health and Welfare (THL), Helsinki, Finland	hannele.mattsson@thl.fi	FinnGen Teams	**Registry Data Operations**
Kati Kristiansson	THL Biobank / Finnish Institute for Health and Welfare (THL), Helsinki, Finland	kati.kristiansson@thl.fi	FinnGen Teams	**Registry Data Operations**
Susanna Lemmelä	Institute for Molecular Medicine Finland (FIMM), HiLIFE, University of Helsinki, Helsink	susanna.lemmela@helsinki.fi	FinnGen Teams	**Registry Data Operations**
Sami Koskelainen	THL Biobank / Finnish Institute for Health and Welfare (THL), Helsinki, Finland	sami.koskelainen@thl.fi	FinnGen Teams	**Registry Data Operations**
Tero Hiekkalinna	THL Biobank / Finnish Institute for Health and Welfare (THL), Helsinki, Finland	tero.hiekkalinna@helsinki.fi	FinnGen Teams	**Registry Data Operations**
Teemu Paajanen	THL Biobank / Finnish Institute for Health and Welfare (THL), Helsinki, Finland	teemu.paajanen@thl.fi	FinnGen Teams	**Registry Data Operations**
Priit Palta	Institute for Molecular Medicine Finland (FIMM), HiLIFE, University of Helsinki, Helsink	priit.palta@helsinki.fi	FinnGen Teams	**Sequencing Informatics**
Shuang Luo	Institute for Molecular Medicine Finland (FIMM), HiLIFE, University of Helsinki, Helsink	shuang.luo@helsinki.fi	FinnGen Teams	**Sequencing Informatics**
Tarja Laitinen	Pirkanmaa Hospital District, Tampere, Finland	tarja.laitinen@pshp.fi	FinnGen Teams	**Trajectory**
Mary Pat Reeve	Institute for Molecular Medicine Finland (FIMM), HiLIFE, University of Helsinki, Helsink	mary.reeve@helsinki.fi	FinnGen Teams	**Trajectory**
Shanmukha Sampath Pa	Institute for Molecular Medicine Finland (FIMM), HiLIFE, University of Helsinki, Helsink	sam.padmanabhuni@helsinki.fi	FinnGen Teams	**Trajectory**
Marianna Niemi	University of Tampere, Tampere, Finland	marianna.niemi@tuni.fi	FinnGen Teams	**Trajectory**
Harri Siirtola	University of Tampere, Tampere, Finland	harri.siirtola@tuni.fi	FinnGen Teams	**Trajectory**
Javier Gracia-Tabuenca	University of Tampere, Tampere, Finland	javier.graciatabuenca@tuni.fi	FinnGen Teams	**Trajectory**
Mika Helminen	University of Tampere, Tampere, Finland	mika.helminen@tuni.fi	FinnGen Teams	**Trajectory**
Tiina Luukkaala	University of Tampere, Tampere, Finland	tiina.luukkaala@tuni.fi	FinnGen Teams	**Trajectory**
Iida Vähätalo	University of Tampere, Tampere, Finland	iida.vahatalo@epshp.fi	FinnGen Teams	**Trajectory**

Jyrki Tammerluoto	Institute for Molecular Medicine Finland (FIMM), HiLIFE, University of Helsinki, Helsink	jyrki.tammerluoto@helsinki.fi	**FinnGen Teams**	**Data protection officer**
Marco Hautalahti	Finnish Biobank Cooperative - FINBB	marco.hautalahti@finbb.fi	**FinnGen Teams**	**FINBB - Finnish biobank cooperative**
Johanna Mäkelä	Finnish Biobank Cooperative - FINBB	johanna.makela@finbb.fi	**FinnGen Teams**	**FINBB - Finnish biobank cooperative**
Sarah Smith	Finnish Biobank Cooperative - FINBB	sarah.smith@finbb.fi	**FinnGen Teams**	**FINBB - Finnish biobank cooperative**
Tom Southerington	Finnish Biobank Cooperative - FINBB	tom.southerington@finbb.fi	**FinnGen Teams**	**FINBB - Finnish biobank cooperative**
Petri Lehto	Finnish Biobank Cooperative - FINBB	petri.lehto@finbb.fi	**FinnGen Teams**	**FINBB - Finnish biobank cooperative**

Estonian Biobank research team

Full Name	Affiliation
	Institute of Genomics, University of Tartu, Estonia
Andres Metspalu	The Institute of Molecular and Cell Biology, University of Tartu, Estonia
Lili Milani	Institute of Genomics, University of Tartu, Estonia
Tõnu Esko	Institute of Genomics, University of Tartu, Estonia
Reedik Mägi	Institute of Genomics, University of Tartu, Estonia
Mari Nelis	Core Facility of Genomics, Institute of Genomics, University of Tartu, Estonia
Georgi Hudjashov	Institute of Genomics, University of Tartu, Estonia

E-mail	Role 1	Role 2
andres.metspalu@ut.ee	sample collection	Head of the EstBB cohort
lili.milani@ut.ee		
tonu.esko@ut.ee		
reedik.magi@ut.ee	QC of the data	
mari.nelis@ut.ee	Sample genotyping	
georgi.hudjashov@ut.ee		
