# Supplementary material for: Polygenic risk scores as a marker for epilepsy risk across lifetime and after unspecified seizure events
Source: Nat Commun. 2024 Jul 25;15:6277. doi: 10.1038/s41467-024-50295-z (PMC11272783; doi:10.1038/s41467-024-50295-z)
Supplement: Supplementary file 3 — Description of Additional Supplementary Files [file 41467_2024_50295_MOESM3_ESM.pdf]

Inventory of Supporting Information:

Supplementary Information: This pdf file contains Supplementary Figures 1-7, Supplementary Tables 1-4 and one Supplementary Note.

Supplementary Data 1: This compressed text file contains the weights used for calculation of PRS_{GGE}, PRS_{NAFE} and PRS_{all}. For each epilepsy GWAS used to calculate the PRSs, we define effect alleles and corresponding weights for the loci, stored in GWAS-specific weight files.

Source Data: This compressed file contains the source data summary statistics tables used to create (parts of) Figures 2, 3 and 4 as tab-separated text files or excel-tables.

FinnGen banner authors: The FinnGen banner contains the full list of FinnGen consortium members.

EstBB banner authors: The Estonian biobank banner contains the full list of Estonian biobank members.
