# Supplementary material for: Polygenic risk scores as a marker for epilepsy risk across lifetime and after unspecified seizure events
Source: Nat Commun. 2024 Jul 25;15:6277. doi: 10.1038/s41467-024-50295-z (PMC11272783; doi:10.1038/s41467-024-50295-z)
Supplement: Supplementary file 5 — Reporting Summary [file 41467_2024_50295_MOESM5_ESM.pdf]

nature portfolio

Corresponding author(s): Henrike O. Heyne

Last updated by author(s): Apr 16, 2024

Reporting Summary

Nature Portfolio wishes to improve the reproducibility of the work that we publish. This form provides structure for consistency and transparency in reporting. For further information on Nature Portfolio policies, see our Editorial Policies and the Editorial Policy Checklist.

Statistics

For all statistical analyses, confirm that the following items are present in the figure legend, table legend, main text, or Methods section.

n/a	Confirmed	
☐	☒	The exact sample size (*n*) for each experimental group/condition, given as a discrete number and unit of measurement
☐	☒	A statement on whether measurements were taken from distinct samples or whether the same sample was measured repeatedly
☐	☒	The statistical test(s) used AND whether they are one- or two-sided<br>*Only common tests should be described solely by name; describe more complex techniques in the Methods section.*
☐	☒	A description of all covariates tested
☐	☒	A description of any assumptions or corrections, such as tests of normality and adjustment for multiple comparisons
☐	☒	A full description of the statistical parameters including central tendency (e.g. means) or other basic estimates (e.g. regression coefficient) AND variation (e.g. standard deviation) or associated estimates of uncertainty (e.g. confidence intervals)
☐	☒	For null hypothesis testing, the test statistic (e.g. *F*, *t*, *r*) with confidence intervals, effect sizes, degrees of freedom and *P* value noted<br>*Give P values as exact values whenever suitable.*
☒	☐	For Bayesian analysis, information on the choice of priors and Markov chain Monte Carlo settings
☒	☐	For hierarchical and complex designs, identification of the appropriate level for tests and full reporting of outcomes
☐	☒	Estimates of effect sizes (e.g. Cohen's *d*, Pearson's *r*), indicating how they were calculated

Our web collection on statistics for biologists contains articles on many of the points above.

Software and code

Policy information about availability of computer code

Data collection	Genotype calls were made with GenCall and zCall algorithms for Illumina and AxiomGT1 algorithm for Affymetrix data. Genotype imputation was done with Beagle 4.1 (version 08Jun17.d8b) with the population-specific SISu v3 reference panel created from high-quality WGS data of 3,775 individuals. Further processing of the genotype data was done using standard genome analysis software Plink 1.9 and 2.0 and BCFtools 1.7 and 1.9.
Data analysis	Pipelines for parallel computing were created using Cromwell-29 and 31 and Wdltool-0.14. Further processing of the data was done using R 3.4.1 (packages: data.table 1.10.4, sm 2.2-5.4). Statistical analyses and figures were done using additional R packages ggplot2, plyr, survminer, survival, tidyr, Rutils.

For manuscripts utilizing custom algorithms or software that are central to the research but not yet described in published literature, software must be made available to editors and reviewers. We strongly encourage code deposition in a community repository (e.g. GitHub). See the Nature Portfolio guidelines for submitting code & software for further information.

Data

Policy information about availability of data

All manuscripts must include a data availability statement. This statement should provide the following information, where applicable:

- Accession codes, unique identifiers, or web links for publicly available datasets
- A description of any restrictions on data availability
- For clinical datasets or third party data, please ensure that the statement adheres to our policy

All results described in this manuscript can be found in the (Supplementary) Tables. A full list of FinnGen endpoints for release 12 is available at https://www.finngen.fi/en/researchers/clinical-endpoints. Individual level data in this study are not publicly available due to legal and privacy limitations, but they can be accessed through individual participating biobanks. The FinnGen data may be accessed through Finnish Biobanks' FinBB portal (www.finbb.fi; email: info.fingenious@finbb.fi). Researchers interested in Estonian Biobank can request access at https://www.geenivaramu.ee/en/access-biobank. For access to data from BioMe biobank, please read here (https://icahn.mssm.edu/research/ipm/programs/biome-biobank). For questions, please reach out to biomebiobank@mssm.edu. Source data in the form of summary statistics are provided with this paper.

Research involving human participants, their data, or biological material

Policy information about studies with human participants or human data. See also policy information about sex, gender (identity/presentation), and sexual orientation and race, ethnicity and racism.

Reporting on sex and gender	Sex-specific analyses were done as indicated in the manuscript.
Reporting on race, ethnicity, or other socially relevant groupings	In all analyses we used birth year as a covariate to account for varying definitions of epilepsy over time. At the same time, this accounts for the influence of age on presence of epilepsy. When not conducting separate analyses in males and females we include sex as a covariate as we show it has effects on epilepsy case status. We ascertain biological sex from genetic analyses. We further include genotyping batch and the first ten PCs of genotypes as covariates to account for potential confounders of batch effect and population structure, respectively.
Population characteristics	520,105 participants of FinnGen are on average 62.1 ± 19 (mean ± standard deviation) years old, 282,064 are female. As samples were mainly collected through legacy collections and hospital biobanks the estimated FinnGen participant may be expected to have more diseases than Finnish population average. Phenotype data comes from digital health record data from Finnish health registries.
Recruitment	The collected samples consist of two entities: 1) legacy samples, mainly collected by the THL (National Institute for Health and Welfare in Finland) and 2) prospective samples which were mainly be collected by hospital biobanks. Almost all of the Finnish biobanks are part of the FinnGen study.
Ethics oversight	The FinnGen project has been approved by the Coordinating Ethics Committee of the Helsinki and Uusimaa Hospital District.

Note that full information on the approval of the study protocol must also be provided in the manuscript.

Field-specific reporting

Please select the one below that is the best fit for your research. If you are not sure, read the appropriate sections before making your selection.

☒ Life sciences ☐ Behavioural & social sciences ☐ Ecological, evolutionary & environmental sciences

For a reference copy of the document with all sections, see nature.com/documents/nr-reporting-summary-flat.pdf

Life sciences study design

All studies must disclose on these points even when the disclosure is negative.

Sample size	All participants of FinnGen datafreeze 12 and Estonian biobank were included in the study. No statistical method was used to predetermine sample size, instead the maximum number of available samples from the respective studies were used.
Data exclusions	Individuals with missing minimum phenotype data or a mismatch between imputed sex and sex in registry data as well as population outliers in PCA have been excluded.
Replication	Results of PRS effects on lifetime epilepsy were successfully validated in Estonian biobank and in BioMe. Validation of individual ancestry groups in BioMe failed due to small sample sizes.
Randomization	In all analyses birth year sex, genotyping batch and the first ten PCs of genotypes were included as covariates.
Blinding	In our analyses investigating effects of PRS on presence of epilepsy, blinding was not possible due to one person conducting analyses alone. However, in an analytical biobank setting, blinding is also rather uncommon.

Reporting for specific materials, systems and methods

We require information from authors about some types of materials, experimental systems and methods used in many studies. Here, indicate whether each material, system or method listed is relevant to your study. If you are not sure if a list item applies to your research, read the appropriate section before selecting a response.

Materials & experimental systems

n/a	Involved in the study
☒	☐ Antibodies
☒	☐ Eukaryotic cell lines
☒	☐ Palaeontology and archaeology
☒	☐ Animals and other organisms
☐	☒ Clinical data
☒	☐ Dual use research of concern
☒	☐ Plants

Methods

n/a	Involved in the study
☒	☐ ChIP-seq
☒	☐ Flow cytometry
☒	☐ MRI-based neuroimaging

Clinical data

Policy information about clinical studies

All manuscripts should comply with the ICMJE guidelines for publication of clinical research and a completed CONSORT checklist must be included with all submissions.

Clinical trial registration	No clinical trial was done.
Study protocol	*Note where the full trial protocol can be accessed OR if not available, explain why.*
Data collection	*Describe the settings and locales of data collection, noting the time periods of recruitment and data collection.*
Outcomes	*Describe how you pre-defined primary and secondary outcome measures and how you assessed these measures.*

Plants

Seed stocks	No research in plants was done.
Novel plant genotypes	*Describe the methods by which all novel plant genotypes were produced. This includes those generated by transgenic approaches, gene editing, chemical/radiation-based mutagenesis and hybridization. For transgenic lines, describe the transformation method, the number of independent lines analyzed and the generation upon which experiments were performed. For gene-edited lines, describe the editor used, the endogenous sequence targeted for editing, the targeting guide RNA sequence (if applicable) and how the editor was applied.*
Authentication	*Describe any authentication procedures for each seed stock used or novel genotype generated. Describe any experiments used to assess the effect of a mutation and, where applicable, how potential secondary effects (e.g. second site T-DNA insertions, mosiacism, off-target gene editing) were examined.*
