# Supplementary material for: Polygenic risk scores as a marker for epilepsy risk across lifetime and after unspecified seizure events
Source: Nat Commun. 2024 Jul 25;15:6277. doi: 10.1038/s41467-024-50295-z (PMC11272783; doi:10.1038/s41467-024-50295-z)
Supplement: Supplementary file 2 — Peer Review File [file 41467_2024_50295_MOESM2_ESM.pdf]

nature portfolio

Peer Review File

Polygenic risk scores as a marker for epilepsy risk across lifetime and after unspecified seizure events

Open Access This file is licensed under a Creative Commons Attribution 4.0 International License, which permits use, sharing, adaptation, distribution and reproduction in any medium or format, as long as you give appropriate credit to the original author(s) and the source, provide a link to the Creative Commons license, and indicate if changes were made. In the cases where the authors are anonymous, such as is the case for the reports of anonymous peer reviewers, author attribution should be to 'Anonymous Referee' followed by a clear attribution to the source work. The images or other third party material in this file are included in the article's Creative Commons license, unless indicated otherwise in a credit line to the material. If material is not included in the article's Creative Commons license and your intended use is not permitted by statutory regulation or exceeds the permitted use, you will need to obtain permission directly from the copyright holder. To view a copy of this license, visit http://creativecommons.org/licenses/by/4.0/.

REVIEWER COMMENTS

Reviewer #1 (Remarks to the Author):

This intriguing study leverages multiple unique longitudinal datasets to explore the potential of common epilepsy genetic risk as a biomarker. The authors conducted comprehensive tests on polygenic risk scores (PRS) for both Generalized Genetic Epilepsy (GGE) and Non-Acquired Focal Epilepsy (NAFE). They offer GGE and NAFE risk estimates across various PRS categories, taking into account different clinical conditions, age groups, and genders. Their findings reveal a significant increase in epilepsy risk among individuals in higher PRS categories, accompanied by several other noteworthy observations.

Furthermore, the authors assert that epilepsy-specific PRSs exhibit strong discriminative capabilities, particularly after the occurrence of a first seizure event. This suggests its potential utility as a clinical biomarker.

Overall, the manuscript is well-written, and the analyses are clear and appropriate. However, I suggest that the authors provide more substantial evidence to support their conclusions and certain claims within the paper.

Comments
1- The main relevance of the paper, in my opinion, would be the clinical utility of the PRS. Certainly, the demonstration that epilepsy PRSs have similar correlations in a longitudinal data set as in 'standard' case control cohort is a valuable observation. However, the main question is how the high risk translates in predictive values such as sensitivity and specificity. I believe this should be provided. In that sense suppl. table 3 is an important table that also provides the number of cases and controls in each PRS bin.
I would like to suggest to include this table in the main text and add percentages and the sensitivity and specificity values.

2- As from above, I think the percentages of cases and controls across the PRS bin show that the PRS alone is not able to serve as a clinical useful biomarker. This observation (provided the authors agree) should be discussed and presented properly in the manuscript.

3- It seemes feasible to extract a 'first seizure cohort' from the records: people that experienced a single seizure versus people that had multiple seizures and ASM use. It would be interesting to see the PRS difference in this group and calculate the predictive values for the PRSs. Would that be possible?

4- The findings on the effect of age is intriguing, but I am a little puzzled as the age categories align with age of onsets. Is the correlation between age and effects reflecting an age effect or difference in diagnostic accuracy?

5- The authors conclude that the GGE PRS is very specific and has potential diagnostic value based on the fact that their PheWAS is largely negative. I am not convinced that this is a valid conclusion, in line with my comment 1, diagnostic utility is defined differently. Furthermore, the lack of significance in the PheWAS may be due to power, and also is at odds with the correlations between epilepsy and other phenotypes described in the latest epilepsy GWAS and the older Brainstorm paper.

6- In the discussion, 3rd aline, it is stated that the PRS effect in GGE is largely driven by females. I believe this is overstating the effect of an HR 1.7 in females versus 1.3 in males. To substantiate this statement a formal regression analysis on sex would be needed?
Furthermore, the statements below confirm that genetic effects are highly correlated between males and females.

Reviewer #2 (Remarks to the Author):

Overall comments

Paper addresses a significant clinical problem: prediction of epilepsy post first time seizure. This is an important problem because seizures can be caused by many (non-genetic) factors such as trauma, illness and it can be hard to know if more seizures are to be expected i.e. a diagnosis of epilepsy is likely. This is important for the safety of the patient. Hence biomarkers are important.

FinnGen is an impressive population cohort with immense power with 370K individuals linked to electronic health records and was used as the discovery cohort. Similarly the Estonian biobank is another important biobank and was used as a validation cohort. The use of these two biobanks (rather than just one) considerably strengthens the paper. The authors also tried to replicate in the more diverse BioMe cohort but were unsuccessful. Attempts to replicate results in non-European ancestries in the BioMe cohort were underpowered. Despite this, it is important that we continue to include diverse ancestries in genetic studies.
However the findings rely on phenotyping (whether GGE or focal) based on what in the electronic health records. The authors have used a combination of ICD coding (clinical) + purchase of anti seizure medication (ASM) to refine their phenotype.

Curiously the authors make use of the old GWAS 2018 summary statistics when the new GWAS summary statistics were released with a pre-print of the manuscript in 2022. It is now accepted (ILAE Consortium 2023, Nat Genet). Hence all the results will have to be regenerated for these new PRS.

The results in this paper also have important repercussions for future studies in epilepsy e.g. GWAS for the usage of EHR defined epilepsy versus clinically-defined epilepsy, with results being very similar.

The sex interaction (p10) for GGE, but not NAFE, is intriguing especially given the absence of signals on the X chromosome. This is an important finding. As the authors note, similar sex differences were not revealed by the 2023 epilepsy sex-specific GWAS performed by the ILAE Consortium on Complex Epilepsies. It will be important to see if the GGE sex effect holds using the 2023 GGE GWAS for PRS generation.

The finding that PRS NAFE is predictive in other diseases/traits, whilst PRS GGE is highly specific, is fascinating and matches thinking in the epilepsy community that this represents a very diverse cohort of diseases not well described as an entity. The findings themselves are somewhat unexpected and will fuel further discussion. Particularly the finding of NAFE and vertebral phenotypes is very intriguing.

Paper is incisively written and easy to read. Analyses are appropriate and graphic representations very powerful (eg. Fig 3). Tables are also well presented.

The paper has novelty in that it is the first time epilepsy PRS has been investigated in the context of a first seizure. This is very important for future clinical translation of PRS. The effect sizes reported across the lifetime are similar to that reported in a similar study in 2019 (Leu et al). That study was applied to a clinically curated cohort, whereas this study relied primarily on ICD codes.

This paper will be of great interest to the epilepsy community and is likely to be well cited as there are immediate follow up studies that these results suggest.

Major points:
• The authors cite the new epilepsy GWAS (although this needs to be updated to the Nat Genet reference), but do not use this larger, more powerful study for the PRS, using instead the previous 2018 GWAS results. This is a missed opportunity. If the reason for this decision was because the FinnGen cohort was included in the 2023 epilepsy GWAS, this should at least be commented on. Strong consideration should be given to obtaining a version of the 2023 epilepsy GWAS sumstats with FinnGen excluded – this would be of great interest to the epilepsy genetics community and

give this paper particular novelty.
• Related to this point, the authors state that none of their cohort contributed to the 2018 epilepsy GWAS used for PRS. Please expand the methods to include how this was determined noting that it appears some case contributions from Finland were made to this 2018 GWAS.
• The authors also state that they did not remove related samples and provide a medRxiv reference supporting this decision. How many cases with epilepsy are related in the FinnGen cohort, and to what degree are they related? Are related pairs/families present in the genetically enriched IGE group? Do results change if the logistic regressions are adjusted for related samples eg including a family identifier as a random effect in a LMM? Further evidence needs to be provided to justify this decision. Since epilepsy has more monogenic drivers it is likely that more relatedness is present.
• Did the authors consider an epilepsy PRS derived from the `all epilepsy` GWAS? This would have the added advantage of it being appliable to all epilepsy diagnoses (i.e., a larger target sample size noting that there are 3,242 patients with "other epilepsy diagnoses" listed in Supp Table 1). An 'all epilepsy' PRS was also not performed by Leu et al 2019 and would be another point of difference.
• Please include the number of SNPs & (p-value threshold) contributing to each epilepsy model investigated as a supplementary table to enable easier comparison to that applied in the Leu et al 2019 study. The inclusion of >835K SNPs as detailed in the text suggests that the models are quite different and should be acknowledged, particularly in the context of reporting similar effect sizes to this earlier study.
• The similar effect sizes achieved in this study, that relied on ICD codes compared to the Leu et al 2019 cohort that was clinically curated, is noted. Can the authors comment further/speculate on the GGE cohort in particular? What are the diagnoses for the non-IGE, GGE patients likely to be? Table 1 and Supplementary Table 4 indicate a late age of onset for the majority of GGE patients (e.g., >20 years). This jumped out at unusual, but perhaps it is expected given the nature of the data available? Please comment further, particularly in the context of the statement made in the abstract that larger effects of GGE PRS are seen in younger age groups.
• Why was the UKBiobank not used as another easily accessible biobank for replication?

Minor Points
• Abstract – please define "high" GGE PRS (e.g., top X-%ile or >X SD) when first mentioned.
• Table 2 – please include sample size Ns.
• The association for the NAFE PRS with back pain is unexpected. Please discuss this in more detail, perhaps in the context of the limitations of these exploratory analyses.
• The main text would benefit from an additional sentence introducing the BioMe cohort e.g., listing the non-European ancestries it includes and cohort sizes, etc.
• P 6 - PRS generated (P6) should detail p-value threshold that was applied.
• P11 - The authors should clarify in the main text that the PheWAS are solely conducted in FinnGen (why not in Estonian biobank?). Note: these could also be conducted in the UKBiobank?
• P13 – Remove "Of course, …"
• P18 & P19 – Please state the definitions of the ICD codes alongside the actual codes.

REVIEWER COMMENTS

Reviewer #1 (Remarks to the Author):

This intriguing study leverages multiple unique longitudinal datasets to explore the potential of common epilepsy genetic risk as a biomarker. The authors conducted comprehensive tests on polygenic risk scores (PRS) for both Generalized Genetic Epilepsy (GGE) and Non-Acquired Focal Epilepsy (NAFE). They offer GGE and NAFE risk estimates across various PRS categories, taking into account different clinical conditions, age groups, and genders. Their findings reveal a significant increase in epilepsy risk among individuals in higher PRS categories, accompanied by several other noteworthy observations.

Furthermore, the authors assert that epilepsy-specific PRSs exhibit strong discriminative capabilities, particularly after the occurrence of a first seizure event. This suggests its potential utility as a clinical biomarker.

Overall, the manuscript is well-written, and the analyses are clear and appropriate. However, I suggest that the authors provide more substantial evidence to support their conclusions and certain claims within the paper.

Comments

1- The main relevance of the paper, in my opinion, would be the clinical utility of the PRS. Certainly, the demonstration that epilepsy PRSs have similar correlations in a longitudinal data set as in 'standard' case control cohort is a valuable observation. However, the main question is how the high risk translates in predictive values such as sensitivity and specificity. I believe this should be provided. In that sense suppl. table 3 is an important table that also provides the number of cases and controls in each PRS bin.
I would like to suggest to include this table in the main text and add percentages and the sensitivity and specificity values.
We added Supplementary Table 3 as Table 3 to the main text and included case numbers, sensitivity and specificity values. Of course, the sensitivity values are very low. As we already discussed in the manuscript earlier, epilepsy PRS is not a suitable screening tool for lifetime epilepsy risk.

2- As from above, I think the percentages of cases and controls across the PRS bin show that the PRS alone is not able to serve as a clinical useful biomarker. This observation (provided the authors agree) should be discussed and presented properly in the manuscript.
We agree that any clinical epilepsy diagnosis should NOT be based on the PRS alone but that PRS should rather be used in conjunction with other tools (such as the EEG) and clinical information and discuss this now hopefully more clearly in the manuscript (lines 297ff).

3- It seemes feasible to extract a 'first seizure cohort' from the records: people that experienced a single seizure versus people that had multiple seizures and ASM use. It would be interesting to see the PRS difference in this group and calculate the predictive values for the PRSs. Would that be possible?

This is basically what we did in paragraph *High PRS is associated with epilepsy across lifetime and after unspecified seizure events*: we extracted a cohort of people that had a single versus multiple seizures and a recorded ASM use. In the group with multiple seizures we also required actual epilepsy diagnosis codes. As predictive values for the effect of PRS, we calculated the C-index.

4- The findings on the effect of age is intriguing, but I am a little puzzled as the age categories align with age of onsets. Is the correlation between age and effects reflecting an age effect or difference in diagnostic accuracy?
We actually meant to explore whether epilepsy PRS effects were present and potentially different in different ages of epilepsy onset. We now reworded it to make that clearer. To account for potential differences in diagnostic accuracy over the years, we included birth year as a covariate for all analyses.

5- The authors conclude that the GGE PRS is very specific and has potential diagnostic value based on the fact that their PheWAS is largely negative. I am not convinced that this is a valid conclusion, in line with my comment 1, diagnostic utility is defined differently. Furthermore, the lack of significance in the PheWAS may be due to power, and also is at odds with the correlations between epilepsy and other phenotypes described in the latest epilepsy GWAS and the older Brainstorm paper.
Agreed that this interpretation is not logical, we reworded that section (lines 317 ff) and now also discuss potential lack of power.

6- In the discussion, 3rd aline, it is stated that the PRS effect in GGE is largely driven by females. I believe this is overstating the effect of an HR 1.7 in females versus 1.3 in males. To substantiate this statement a formal regression analysis on sex would be needed?
Furthermore, the statements below confirm that genetic effects are highly correlated between males and females.
We toned down the statement about the PRS effect driven by females in the discussion now reading “the effect of PRS_{GGE} on GGE was substantially larger in females than males”.
Initially, we searched and identified a significant interaction of PRS and sex in a cox model with lifetime epilepsy as a response variable. That prompted us to investigate PRS effect sizes separately in males and females.
As suggested, in a formal regression analysis on sex we also found a significant effect of sex on presence of GGE (n=924, p-value = 0.01) as well as a significant age*sex interaction (p-value = 0.02). When visualizing the effect of age and sex on epilepsy there is a higher prevalence of epilepsy in females, specifically with onset in the teenage - young adult range (see left upper panel in violin plot below).

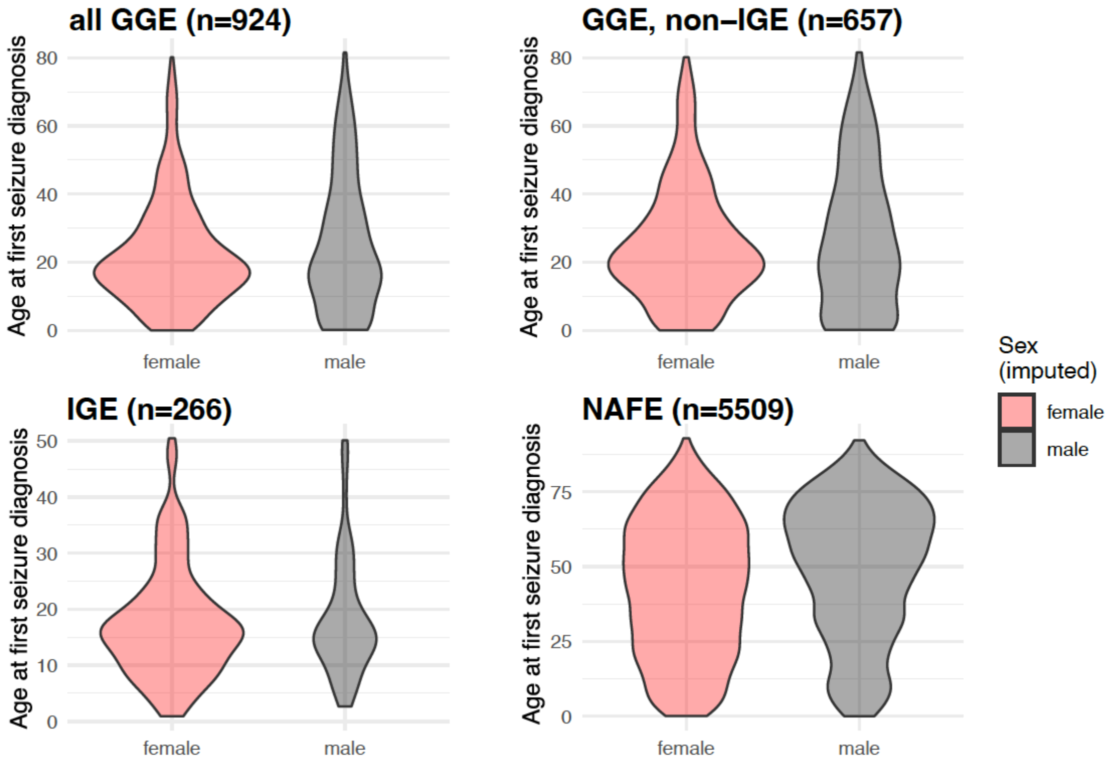

We were thus wondering whether there were sex specific effects on specific epilepsy types. We found indeed a more significant effect of sex (p-value 7x10e-4) and of PRS*sex interaction (1x10e-3) influencing presence of non-IGE GGE (n=657) but no significant effect of sex (p=0.4) nor PRS*sex interaction (p=0.7) on IGE (n=226). Similarly, the effect of GGE-PRS on non-IGE GGE was substantially higher in females (p-value 5x10e-26, HR per SD PRS 1.78, 95% CI 1.60-1.99) than males (p-value 2x10e-5, HR of 1.32 per SD PRS, 95% CI 1.16-1.50) while they were quite comparable in in IGE: females (p-value 1x10e-25, HR per SD PRS 2.35, 95% CI 1.16-1.60; males: p-value 2x10e-10, HR per SD PRS 2.57, 95% CI 1.92-3.44).

In the discussion, we state that genetic effects on GGE have been shown to be highly correlated between males and females but now additionally add that "…we find sex specific PRS effects predominantly for non-IGE suggesting sex-specific genetic factors may differentially influence risk for specific epilepsy subtypes. Thus, further research is needed to elucidate how genetic factors may differently influence epilepsy between sexes."

Reviewer #2 (Remarks to the Author):

Overall comments

Paper addresses a significant clinical problem: prediction of epilepsy post first time seizure. This is an important problem because seizures can be caused by many (non-genetic) factors such as trauma, illness and it can be hard to know if more seizures are to be expected i.e. a diagnosis of epilepsy is likely. This is important for the safety of the patient. Hence biomarkers are important.

FinnGen is an impressive population cohort with immense power with 370K individuals linked to electronic health records and was used as the discovery cohort. Similarly the Estonian biobank is another important biobank and was used as a validation cohort. The use of these two biobanks (rather than just one) considerably strengthens the paper. The authors also tried to replicate in the more diverse BioMe cohort but were unsuccessful. Attempts to replicate results in non-European ancestries in the BioMe cohort were underpowered. Despite this, it is important that we continue to include diverse ancestries in genetic studies.
However the findings rely on phenotyping (whether GGE or focal) based on what in the electronic health records. The authors have used a combination of ICD coding (clinical) + purchase of anti seizure medication (ASM) to refine their phenotype.

Curiously the authors make use of the old GWAS 2018 summary statistics when the new GWAS summary statistics were released with a pre-print of the manuscript in 2022. It is now accepted (ILAE Consortium 2023, Nat Genet). Hence all the results will have to be regenerated for these new PRS.
We regenerated all results with the newest ILAE epilepsy GWAS 2023. It was a lot of work, but seems to have been worth it as the results expectedly improved (see answers below) in line with the increased heritability estimates of the newer GWAS.

The results in this paper also have important repercussions for future studies in epilepsy e.g. GWAS for the usage of EHR defined epilepsy versus clinically-defined epilepsy, with results being very similar.

The sex interaction (p10) for GGE, but not NAFE, is intriguing especially given the absence of signals on the X chromosome. This is an important finding. As the authors note, similar sex differences were not revealed by the 2023 epilepsy sex-specific GWAS performed by the ILAE Consortium on Complex Epilepsies. It will be important to see if the GGE sex effect holds using the 2023 GGE GWAS for PRS generation.
Repeating results with the newest ILAE epilepsy GWAS 2023 and larger data we still found the significant PRS x SEX interaction when estimating PRS effects on lifetime GGE. We also found the different PRS' effect sizes in males versus females. Actually, we now also find a smaller, but significant PRS x SEX interaction on lifetime NAFE.

The finding that PRS NAFE is predictive in other diseases/traits, whilst PRS GGE is highly specific, is fascinating and matches thinking in the epilepsy community that this represents a very diverse cohort of diseases not well described as an entity. The findings themselves are somewhat unexpected and will fuel further discussion. Particularly the finding of NAFE and vertebral phenotypes is very intriguing.

Paper is incisively written and easy to read. Analyses are appropriate and graphic representations very powerful (eg. Fig 3). Tables are also well presented.

The paper has novelty in that it is the first time epilepsy PRS has been investigated in the context of a first seizure. This is very important for future clinical translation of PRS. The effect sizes reported across the lifetime are similar to that reported in a similar study in 2019 (Leu et al). That study was applied to a clinically curated cohort, whereas this study relied primarily on ICD codes.

This paper will be of great interest to the epilepsy community and is likely to be well cited as there are immediate follow up studies that these results suggest.

Major points:

• The authors cite the new epilepsy GWAS (although this needs to be updated to the Nat Genet reference), but do not use this larger, more powerful study for the PRS, using instead the previous 2018 GWAS results. This is a missed opportunity. If the reason for this decision was because the FinnGen cohort was included in the 2023 epilepsy GWAS, this should at least be commented on. Strong consideration should be given to obtaining a version of the 2023 epilepsy GWAS sumstats with FinnGen excluded – this would be of great interest to the epilepsy genetics community and give this paper particular novelty.

We generated all results with the newest ILAE epilepsy GWAS 2023 (and updated the reference). This caused our prediction results to improve substantially. In addition, we also used the newest FinnGen data release 12 increasing our sample size from 350k to 500k. We thus improved predictions to an HR of 1.73 per SD PRS-GGE (p-value 6x10e-43, in comparison with the ILAE 2016 GWAS where we got an HR of 1.6 per SD PRS-GGE). This fits nicely with an increase in GGE heritability from 32% to 40% in the latest GWAS.

FYI: The reason for not using the ILAE 2023 epilepsy GWAS initially was because the first version of the paper was already written before the new GWAS came out. So to be clear, the FinnGen cohort was NOT included in the newest 2023 GWAS cases, however there was some overlap regarding controls, please see comment below.

• Related to this point, the authors state that none of their cohort contributed to the 2018 epilepsy GWAS used for PRS. Please expand the methods to include how this was determined noting that it appears some case contributions from Finland were made to this 2018 GWAS.

We initially explored potential cohort overlaps with the help of one co-author who was involved in both studies. We also double-checked all legacy cohorts part of FinnGen and Finnish cohorts contributing to the ILAE 2023 GWAS. Contributing to the ILAE 2018 paper and the Epi25 Collaborative which formed the largest part of the ILAE 2023 GWAS was the *GenEpa* cohort (n=715 with cases and controls, not part of FinnGen). In addition, the FinRisk cohort (n=40,000 controls) contributed to the ILAE 2023 GWAS. We therefore excluded 25405 FinRisk samples from the controls when using the ILAE 2023 GWAS. We mention this in the manuscript (560ff).

• The authors also state that they did not remove related samples and provide a medRxiv reference supporting this decision. How many cases with epilepsy are related in the FinnGen cohort, and to what degree are they related? Are related pairs/families present in the genetically enriched IGE group? Do results change if the logistic regressions are adjusted for related samples eg including a family identifier as a random effect in a LMM? Further evidence needs to be provided to justify this decision. Since epilepsy has more monogenic drivers it is likely that more relatedness is present.

Using the king software, we identified 320,226 individuals related > 3rd degree (corresponding to kinship values > 0.04) in the FinnGen data corresponding to 62%

of the cohort. Within FinnGen, these related individuals were however heavily enriched in particular batches such as family studies TWINS, FINRISK or COROGENE. Contrary to expectation, we thus found much fewer related individuals (IGE: 6 related out of 226, GGE: 18 related out of 924) in the group of individuals with GGE and IGE. Expectedly, the effect sizes of PRS on lifetime GGE risk (cox model, for IGE: HR of 2.39 per SD PRS, 95%-CI 2.1-2.7, p-value 2x10e-34, for GGE: HR of 1.74 per SD PRS, 95%-CI 1.6-1.9, p-value 1x10e-53) are almost identical when excluding related individuals (even though the control cohort gets markedly smaller). We include a summary of this in the manuscript.
As suggested, we also included a kinship matrix as a random effect in a LMM testing the effect of PRS on epilepsy case status and compared it to a linear model without the kinship matrix. When comparing the two models with an anova we thus found no significant difference (Chi- square 0.27, p-value 0.6, degree of freedom 1).

• Did the authors consider an epilepsy PRS derived from the `all epilepsy` GWAS? This would have the added advantage of it being appliable to all epilepsy diagnoses (i.e., a larger target sample size noting that there are 3,242 patients with “other epilepsy diagnoses” listed in Supp Table 1). An ‘all epilepsy’ PRS was also not performed by Leu et al 2019 and would be another point of difference.
We tested analyses with an all-epilepsy PRS. Unfortunately, we found only limited performance on

- lifetime epilepsy (at least 2 epilepsy codes, n=9,313) (HR of 1.102 per SD PRS, 95%-CI 1.08-1.13, p-value 5x10e-19).
- lifetime GGE risk (HR of 1.03 per SD PRS, 95%-CI 0.96-1.10, p-value 0.4),
- lifetime NAFE risk (HR of 1.12 per SD PRS, 95%-CI 1.09-1.16, p-value 1x10e-16) or

We included these results in the manuscript as a Supplementary Table 3.

• Please include the number of SNPs & (p-value threshold) contributing to each epilepsy model investigated as a supplementary table to enable easier comparison to that applied in the Leu et al 2019 study. The inclusion of >835K SNPs as detailed in the text suggests that the models are quite different and should be acknowledged, particularly in the context of reporting similar effect sizes to this earlier study.
We included a comparison to the Leu et al study regarding the number of included SNPs (lines 555 ff).
“Importantly, we find very similar respective effect sizes of PRS-GGE and PRS-NAFE on GGE and NAFE as reported in previous cohorts from Epi25 or the Cleveland Clinic [24] when using the same GWAS [12] in a previous version of the manuscript [31] or the updated GWAS [13] (see Table 2). We acknowledge, that PRS effects in our cohort may not be directly comparable as we are using a different PRS calculation method (using all weighted 835K SNPs [47] instead of classic clumping and using SNPs < p-value threshold 0.5 [24]). However, differences in phenotype definitions have been reported to have larger effects than differences in PRS methods [48], specifically for epilepsy [13]. We thus consider it likely that the epilepsy phenotypes in our biobank data are comparable to the phenotypes curated according to clinical criteria in these cohorts.”
We now also provide the weight files of our updated PRS analyses to increase reproducibility.

• The similar effect sizes achieved in this study, that relied on ICD codes compared to the Leu et al 2019 cohort that was clinically curated, is noted. Can the authors comment further/speculate on the GGE cohort in particular? What are the diagnoses for the non-IGE, GGE patients likely to be? Table 1 and Supplementary Table 4 indicate a late age of onset for the majority of GGE patients (e.g., >20 years). This jumped out at unusual, but perhaps it is expected given the nature of the data available? Please comment further, particularly in the context of the statement made in the abstract that larger effects of GGE PRS are seen in younger age groups.
Non-IGE, GGE patients include patients who have been diagnosed late with IGE and thus do not anymore present with the classical phenotype of IGE. This group also includes epilepsies starting in young adults with generalized seizures without focal features and without focal findings in EEG or imaging.

• Why was the UKBiobank not used as another easily accessible biobank for replication?
Due to the nature of the different recruitment, participants of the UK biobank are expected to be on average older than in FinnGen which makes it a difficult choice for studying a disease with main onset in childhood or young adhulthood. We thus found only 121 individuals with onset of GGE < 40 years old in the UK biobank and none with onset < 20 years. Therefore, we used the Estonian biobank as a validation cohort instead that also included younger individuals.

Minor Points
• Abstract – please define “high” GGE PRS (e.g., top X-%ile or >X SD) when first mentioned.
Done. (Due to the quantitative nature, we used ‘increasing’ PRS)

• Table 2 – please include sample size Ns.
Done.

• The association for the NAFE PRS with back pain is unexpected. Please discuss this in more detail, perhaps in the context of the limitations of these exploratory analyses.
We briefly discussed the association with back pain and associated psychiatric diseases.

• The main text would benefit from an additional sentence introducing the BioMe cohort e.g., listing the non-European ancestries it includes and cohort sizes, etc.
We added an introduction to the BioMe cohort.

• P 6 - PRS generated (P6) should detail p-value threshold that was applied.
We actually did not use a p-value threshold but instead used a method (PRS-CS) that weights SNPs according to their p-value of association. We explained that now more clearly in the methods section (lines 569 ff).

• P11 - The authors should clarify in the main text that the PheWAS are solely conducted in FinnGen (why not in Estonian biobank?). Note: these could also be conducted in the UKBiobank?

There is a paper that does a PheWAS in the UK biobank that we are citing and referring to, which is why we did the analysis only in FinnGen. Since that analysis is rather exploratory and not central to the paper we did not conduct it in the Estonian biobank.

• P13 – Remove “Of course, …”
Done.

• P18 & P19 – Please state the definitions of the ICD codes alongside the actual codes.
Done.

REVIEWERS' COMMENTS

Reviewer #1 (Remarks to the Author):

The authors have answered all my questions and responded excellently to my comments. I have no further comments.

Reviewer #1 (Remarks on code availability):

n.a.

Reviewer #2 (Remarks to the Author):

The authors have done a substantial amount of work to address original feedback, including updating the results with the latest epilepsy GWAS from 2023. The impact and relevance of this manuscript has been greatly improved as a result.

Major point
Methods:
Please clarify how you determined that there were no sample duplicates or close relatives included in both the discovery GWAS and FinnGen in the manuscript text. Was this done genetically (e.g., by kinship analysis) or by cross-referencing sample IDs? If the latter, please include this as a potential limitation in the Discussion. Inclusion of even a small number of duplicate samples or close relatives has the potential to significantly inflate PRS results (see Wray, N.R., et al. Pitfalls of predicting complex traits from SNPs. Nat Rev Genet 2013).

In particular there is a concern about the bimodal distribution (2ndary smaller peak) in the epilepsy PRS distribution displayed in Fig 1B which may be driven by this.

Minor point
Abstract:
Suggest changing the following sentence to clarify that "younger groups" are with regards to younger age of first seizure or epilepsy onset. As currently written, it is a bit ambiguous: "We further report significantly larger effects of PRS-GGE on epilepsy in females and in younger groups".

The request to remove
"Of course…" from last paragraph on p13 in the original manuscript was reported as corrected in the rebuttal but inspection of the updated manuscript reveals that this has been retained and is now line 319 p 14 in the updated manuscript.

REVIEWER COMMENTS

Reviewer #1 (Remarks to the Author):

The authors have answered all my questions and responded excellently to my comments. I have no further comments.

Reviewer #1 (Remarks on code availability):

n.a.

Reviewer #2 (Remarks to the Author):

The authors have done a substantial amount of work to address original feedback, including updating the results with the latest epilepsy GWAS from 2023. The impact and relevance of this manuscript has been greatly improved as a result.

Major point
Methods:
Please clarify how you determined that there were no sample duplicates or close relatives included in both the discovery GWAS and FinnGen in the manuscript text. Was this done genetically (e.g., by kinship analysis) or by cross-referencing sample IDs? If the latter, please include this as a potential limitation in the Discussion. Inclusion of even a small number of duplicate samples or close relatives has the potential to significantly inflate PRS results (see Wray, N.R., et al. Pitfalls of predicting complex traits from SNPs. Nat Rev Genet 2013).

In particular there is a concern about the bimodal distribution (2ndary smaller peak) in the epilepsy PRS distribution displayed in Fig 1B which may be driven by this.

Unfortunately, we did not have the option to compare individual-level data of GWAS and FinnGen cohort. We have included this as a limitation in the discussion.

Minor point
Abstract:
Suggest changing the following sentence to clarify that “younger groups” are with regards to younger age of first seizure or epilepsy onset. As currently written, it is a bit ambiguous: “We further report significantly larger effects of PRS-GGE on epilepsy in females and in younger groups”.

We clarified this in the abstract.

The request to remove
“Of course…” from last paragraph on p13 in the original manuscript was reported as corrected in the rebuttal but inspection of the updated manuscript reveals that this has been retained and is now line 319 p 14 in the updated manuscript.

We apologize for the mishap and have indeed removed it now (replacing it with “however”).
